# Supplementary material for: Comparative immunogenic and structural analysis of virus-like particle and inactivated whole-virion vaccines against enterovirus D68
Source: Mol Ther Nucleic Acids. 2026 May 20;37(2):102957. doi: 10.1016/j.omtn.2026.102957 (PMC13264187; doi:10.1016/j.omtn.2026.102957)
Supplement: Document S1. Figures S1–S11 and Table S1 [file mmc1.pdf]

OMTN, Volume *37*

Supplemental information

Comparative immunogenic and structural analysis of virus-like particle and inactivated whole-virion vaccines against enterovirus D68

Kota Senpuku, Yuta Kunishima, Mika Hirose, Tatsuya Karaki, Kotaro Taniguchi, Chikako Kataoka-Nakamura, Toshiro Hirai, Koubun Yasuda, Etsushi Kuroda, Takayuki Kato, Taiki Ito, and Yasuo Yoshioka

A

	Database	Accession	Score	Mass	Num. of matches	Num. of significant matches	Num. of sequences	Num. of significant sequences	emPAI	Description
**1**	SwissProt	HS90B_HUMAN	4632	83554	201	201	45	45	12.84	Heat shock protein HSP 90-beta
**2**	SwissProt	HS90A_HUMAN	4315	85006	196	196	43	43	9.02	Heat shock protein HSP 90-alpha
**3**	SwissProt	HS71A_HUMAN	1461	70294	70	70	19	19	2.48	Heat shock 70 kDa protein 1A
**4**	SwissProt	EF2_HUMAN	848	96246	55	55	22	22	1.77	Elongation factor 2
**5**	SwissProt	IMB1_HUMAN	785	98420	39	39	16	16	1.28	Importin subunit beta-1
**6**	SwissProt	K2C1_HUMAN	623	66170	32	32	16	16	1.92	Keratin, type II cytoskeletal 1
**7**	SwissProt	UBA1_HUMAN	590	118858	31	31	14	14	0.62	Ubiquitin-like modifier-activating enzyme 1
**8**	SwissProt	TNPO1_HUMAN	536	103771	29	29	14	14	0.63	Transportin-1
**9**	SwissProt	MCM5_HUMAN	492	83031	31	31	18	18	1.26	DNA replication licensing factor MCM5
**10**	SwissProt	MCM2_HUMAN	481	102516	32	32	17	17	0.93	DNA replication licensing factor MCM2

B

	Database	Accession	Score	Mass	Num. of matches	Num. of significant matches	Num. of sequences	Num. of significant sequences	emPAI	Description
**1**	SwissProt	HS71A_HUMAN	7451	70294	350	350	38	38	25.78	Heat shock 70 kDa protein 1A
**2**	SwissProt	HSP7C_HUMAN	2064	71082	106	106	23	23	6.1	Heat shock cognate 71 kDa protein
**3**	SwissProt	HSP72_HUMAN	1160	70263	53	53	13	13	1.27	Heat shock-related 70 kDa protein 2
**4**	SwissProt	XRCC6_HUMAN	788	70084	40	40	16	16	1.28	X-ray repair cross-complementing protein 6
**5**	SwissProt	PPCE_HUMAN	727	81560	43	43	21	21	2.08	Prolyl endopeptidase
**6**	SwissProt	HS90B_HUMAN	699	83554	39	39	19	19	1.45	Heat shock protein HSP 90-beta
**7**	SwissProt	K2C1_HUMAN	664	66170	34	34	15	15	1.65	Keratin, type II cytoskeletal 1
**8**	SwissProt	SYRC_HUMAN	659	76129	41	41	22	22	2.19	Arginine--tRNA ligase, cytoplasmic
**9**	SwissProt	AMPB_HUMAN	588	73234	32	32	17	17	1.2	Aminopeptidase B
**10**	SwissProt	SNX2_HUMAN	581	58549	36	36	15	15	2.38	Sorting nexin-2

Figure S1. Identification of proteins via LC-MS/MS analysis. LC-MS/MS analysis was performed on the protein bands marked in red in Figure 1C, approximately corresponding to (A) 90 kDa and (B) 70 kDa. The table lists the top 10 candidate proteins identified. emPAI: Exponentially modified protein abundance Index.

A

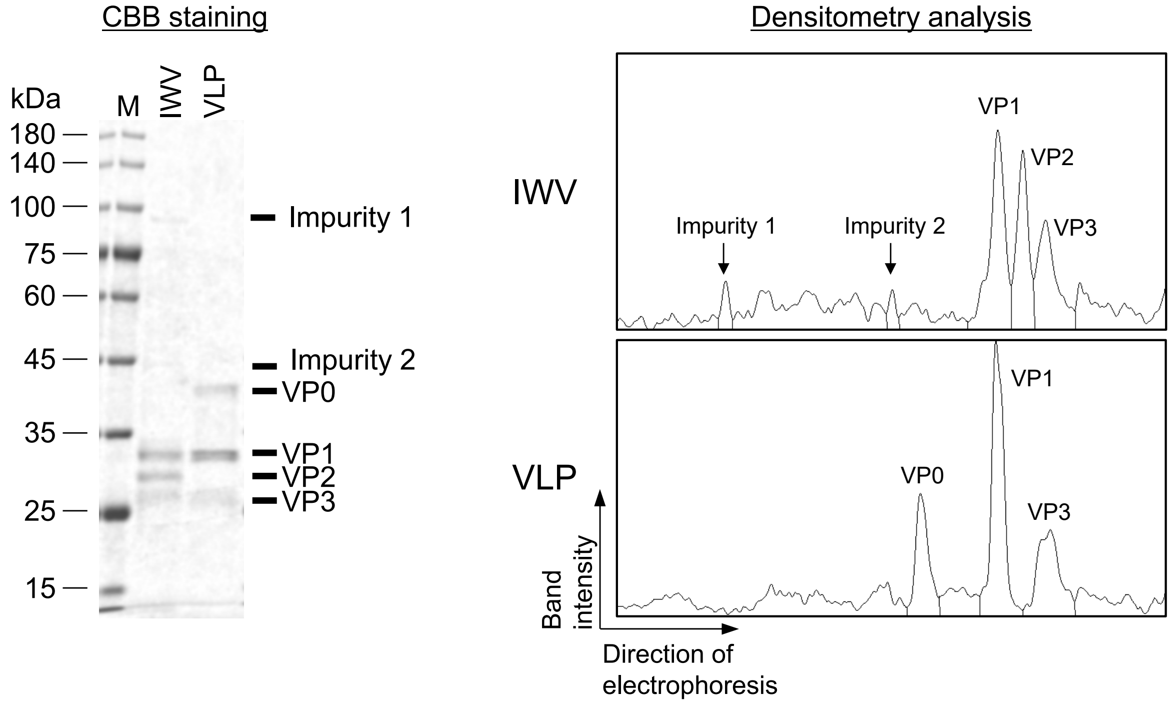

B

IWV		VLP	
Band	Intensity	Band	Intensity
VP1	5759	VP1	6524
VP2	4022	VP0	3104
VP3	3920	VP3	3756
Impurity 1	711		
Impurity 2	552		
Total	14964	Total	13384
VP1-3 total	13701	V0-3 total	13384
Purity	91.5%	Purity	100%

Figure S2. Purity of IWV and VLP used for the vaccination. (A) Purified IWV and VLP (1 μg/lane) were subjected to SDS-PAGE followed by Coomassie Brilliant Blue (CBB) staining. The picture of the gel after CBB staining is shown in the left panel. The detected bands are labeled accordingly. M indicates molecular size marker. The results of the quantitative densitometric analysis for each lane are shown in the right panel. (B) The table showing the result of the band quantification based on the result of the densitometry analysis (right panel of A). Data are representative of at least two independent preparations.

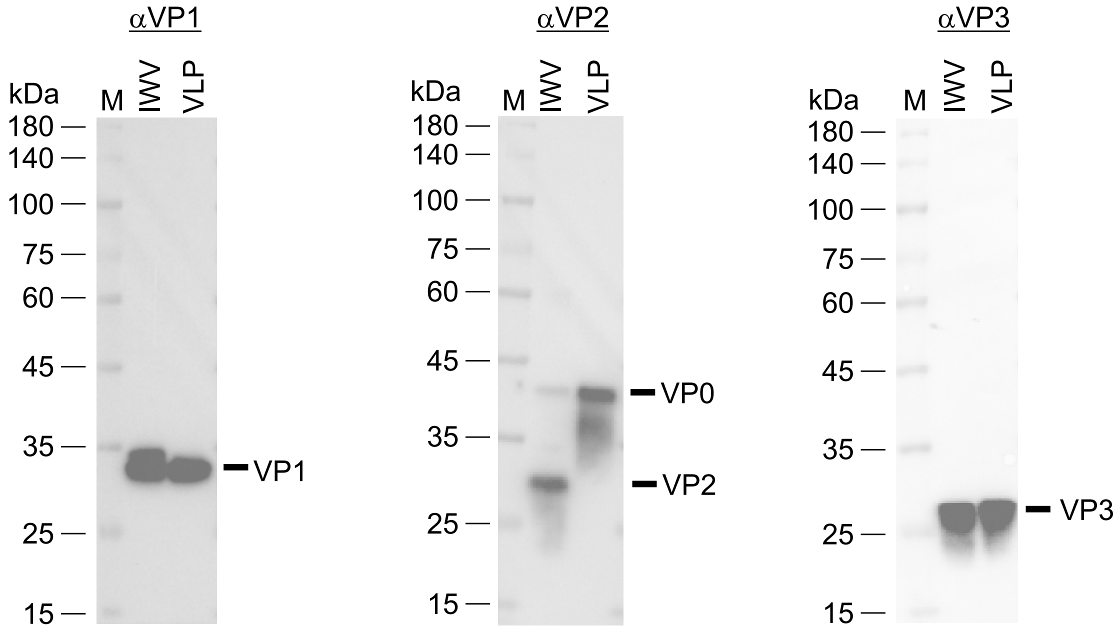

Figure S3. Western blot analysis of purified IWV and VLP. Purified IWV and VLP samples (50 ng/lane) were subjected to western blotting and probed with antibodies specific to VP1, VP2, and VP3 to confirm the presence of capsid proteins. M indicates molecular size marker.

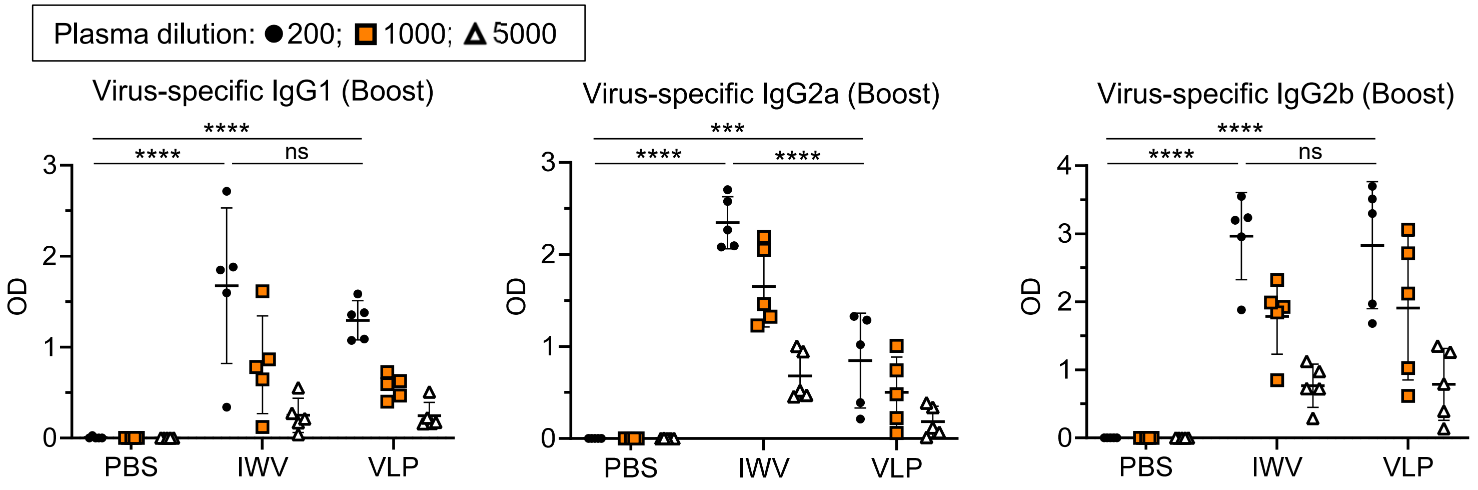

Figure S4. IgG subclass profiles of EV-D68-specific antibodies induced by IWV and VLP. Post-boost plasma levels of virus-specific IgG1, IgG2a, and IgG2b were measured. Data represent the mean ± SD (n = 5 per group). Statistical analyses were performed using 200-fold-diluted plasma samples. "ns" indicates no significant difference. ***$P < 0.001$; ****$P < 0.0001$ as determined by Tukey's test.

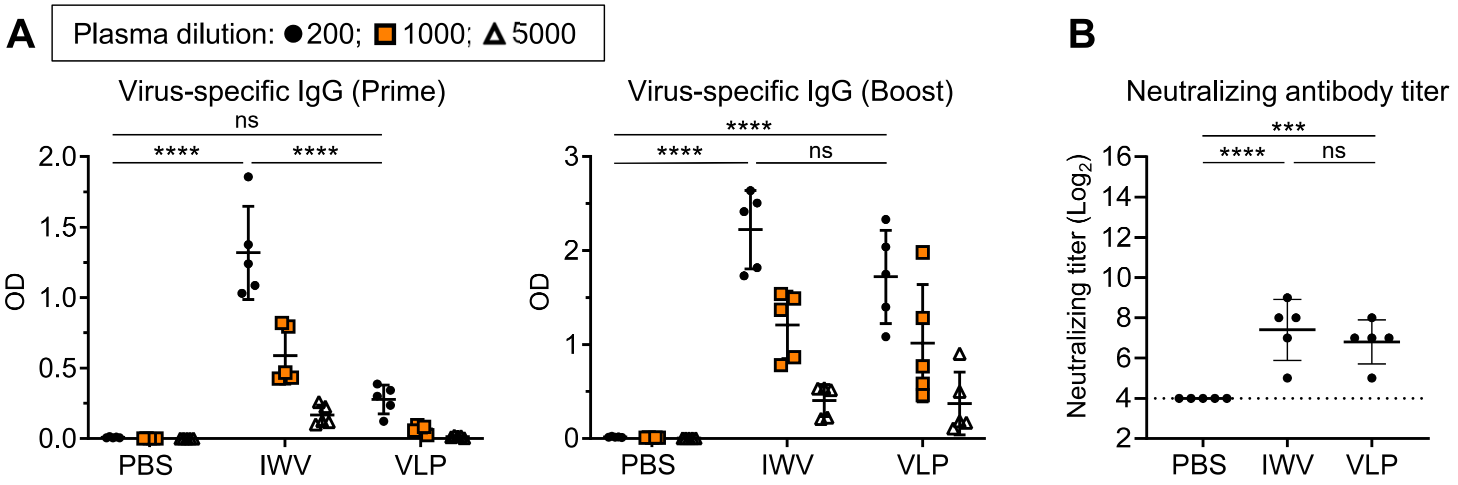

Figure S5. Immunogenicity of IWV and VLP vaccines following intramuscular immunization. Female BALB/c mice were immunized intramuscularly with 0.1 µg of either IWV or VLP on days 0 and 21. (A) Plasma IgG levels specific to IWV following prime and boost immunization. (B) Neutralizing antibody titers against the MO strain measured after boost immunization. (A, B) Each group consisted of n = 5 mice. Data are presented as mean ± SD. (B) Dotted lines represent the limit of detection. (A) Statistical analyses were performed using plasma diluted 1:200. (A, B) "ns" indicates not significant. ***P < 0.001; ****P < 0.0001, as determined by Tukey's test.

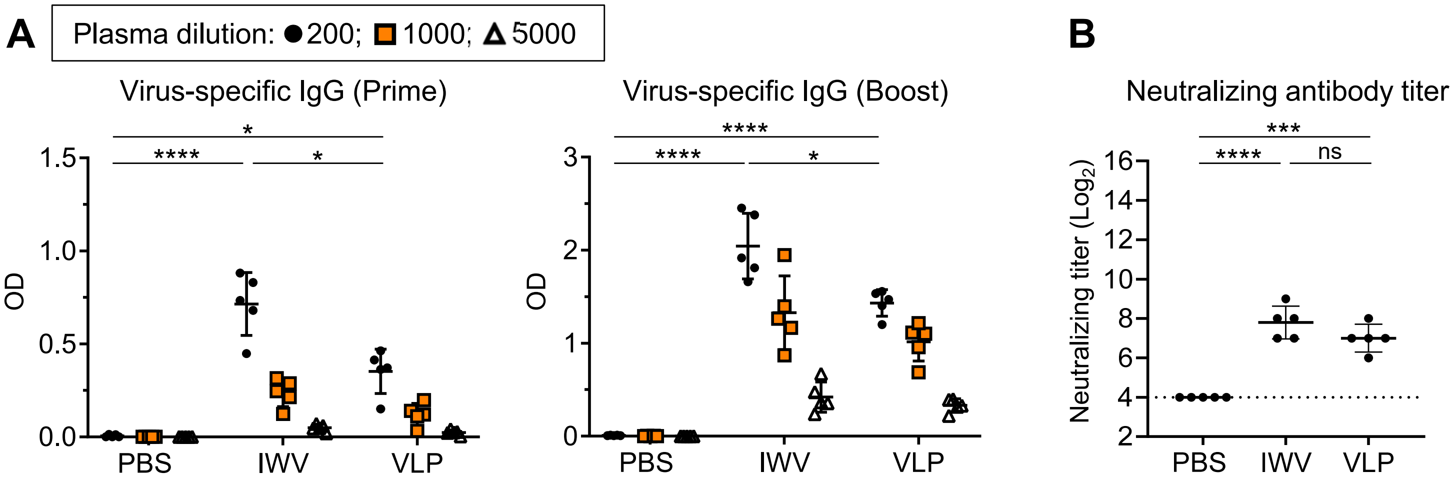

Figure S6. Immunogenicity of IWV and VLP vaccines in male BALB/c mice. Male BALB/c mice were immunized subcutaneously with 0.1 μg of either IWV or VLP on days 0 and 21. (A) Plasma IgG levels specific to IWV following prime and boost immunization. (B) Neutralizing antibody titers against the MO strain measured after boost immunization. (A, B) Each group consisted of n = 5 mice. Data are presented as mean ± SD. (B) Dotted lines represent the limit of detection. (A) Statistical analyses were performed using plasma diluted 1:200. (A, B) “ns” indicates not significant. **P* < 0.05; ****P* < 0.001; *****P* < 0.0001, as determined by Tukey’s test.

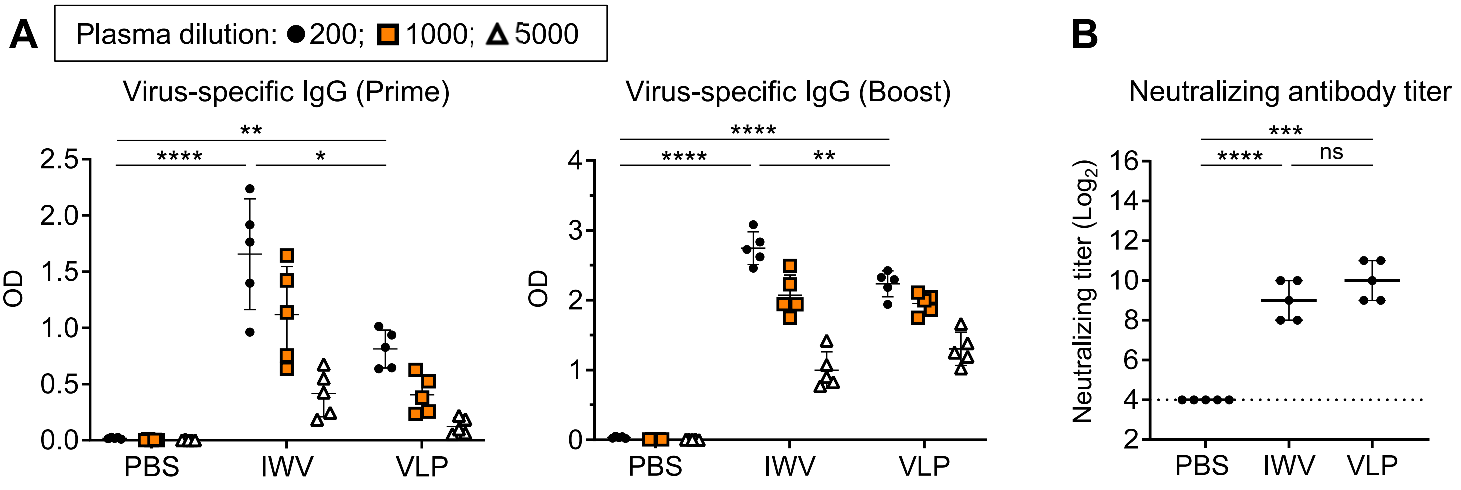

Figure S7. Immunogenicity of IWV and VLP vaccines in female CB6F1 mice. Female CB6F1 mice were immunized subcutaneously with 0.1 μg of either IWV or VLP on days 0 and 21. (A) Plasma IgG levels specific to IWV following prime and boost immunization. (B) Neutralizing antibody titers against the MO strain measured after boost immunization. (A, B) Each group consisted of n = 5 mice. Data are presented as mean ± SD. (B) Dotted lines represent the limit of detection. (A) Statistical analyses were performed using plasma diluted 1:200. (A, B) "ns" indicates not significant. **P* < 0.05; ***P* < 0.01; ****P* < 0.001; *****P* < 0.0001, as determined by Tukey's test.

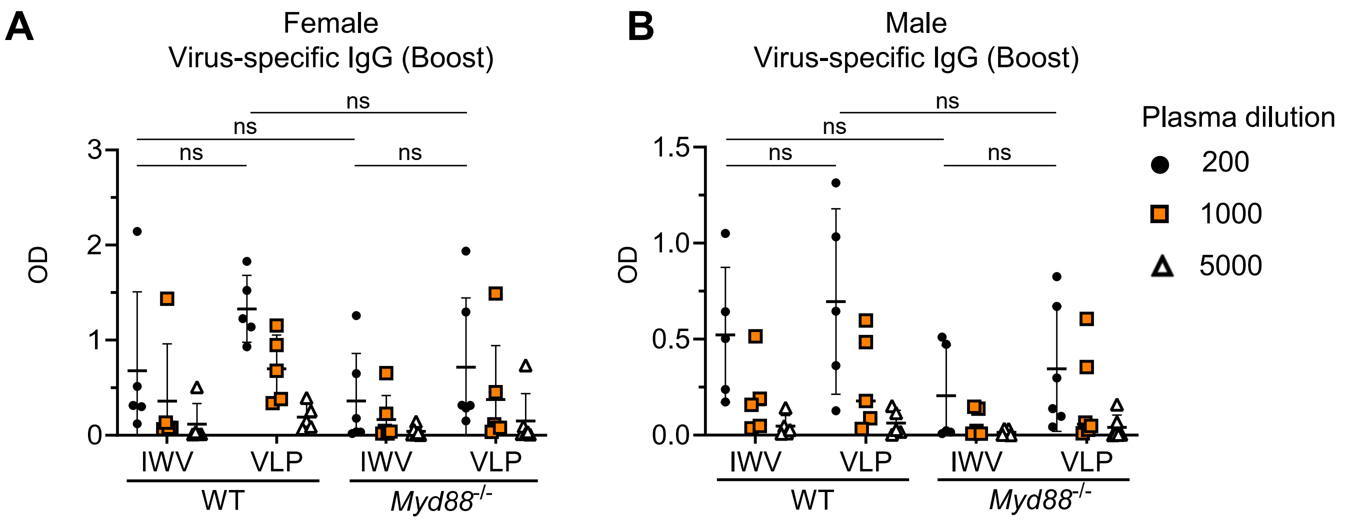

Figure S8. MyD88-independent immune responses induced by IWV and VLP vaccines. (A) Female and (B) male wild-type (WT) and MyD88-deficient (*Myd88*$^{-/-}$) mice were immunized subcutaneously with 0.1 μg of IWV or VLP on days 0 and 21. Virus-specific IgG levels in plasma from (A) female and (B) male mice following boost immunization were shown. (A, B) Each group consisted of n = 5 or 6 mice. Data are presented as mean ± SD. Statistical analyses were performed using plasma diluted 1:200. “ns” indicates not significant, as determined by Tukey’s test.

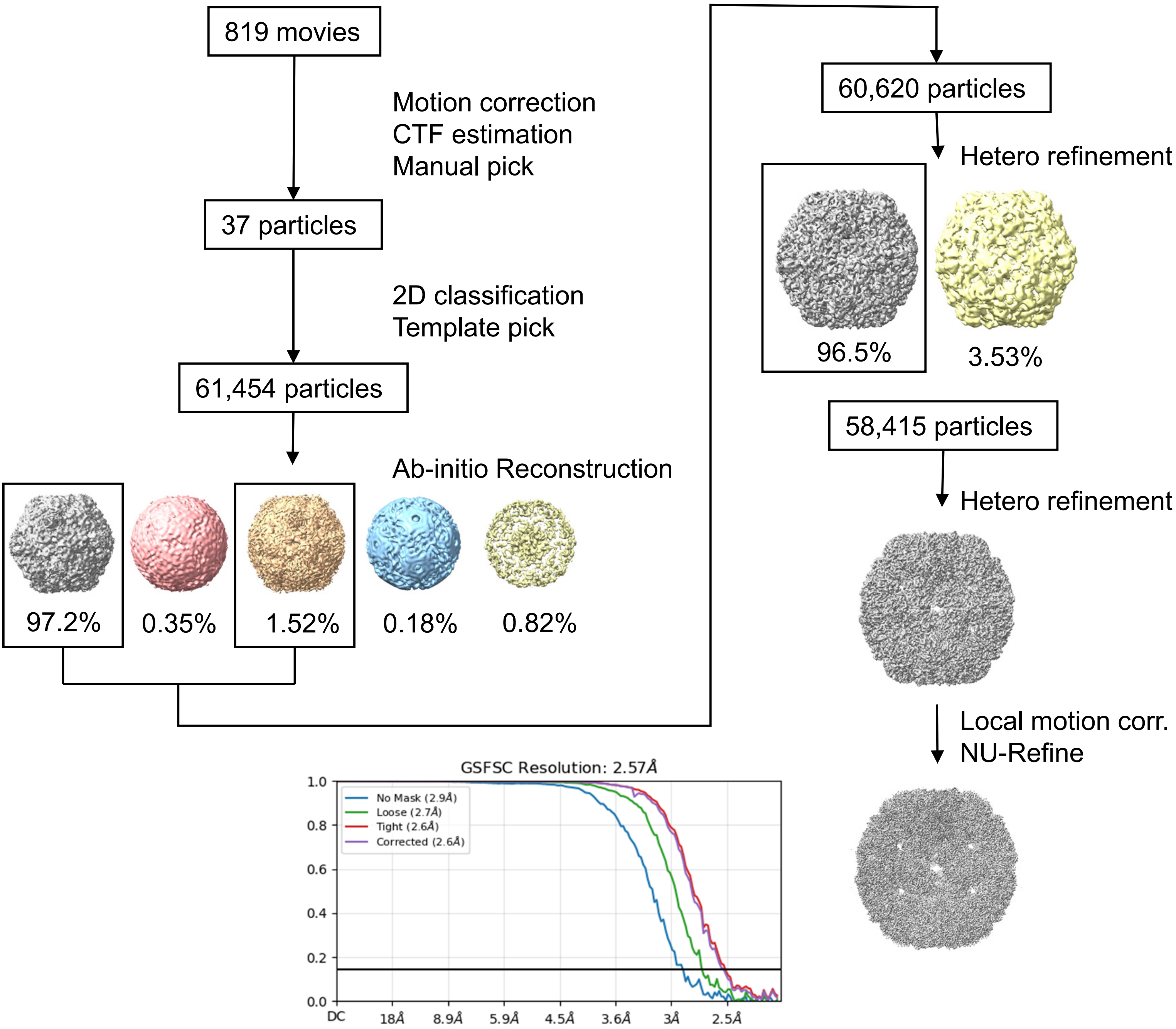

Figure S9. Workflow of cryo-EM image processing. Overall workflow of image processing using cryoSPARC version 4.6.0 software. Gold-standard Fourier shell correlation curves of the reconstructed cryo-EM maps are shown in the bottom.

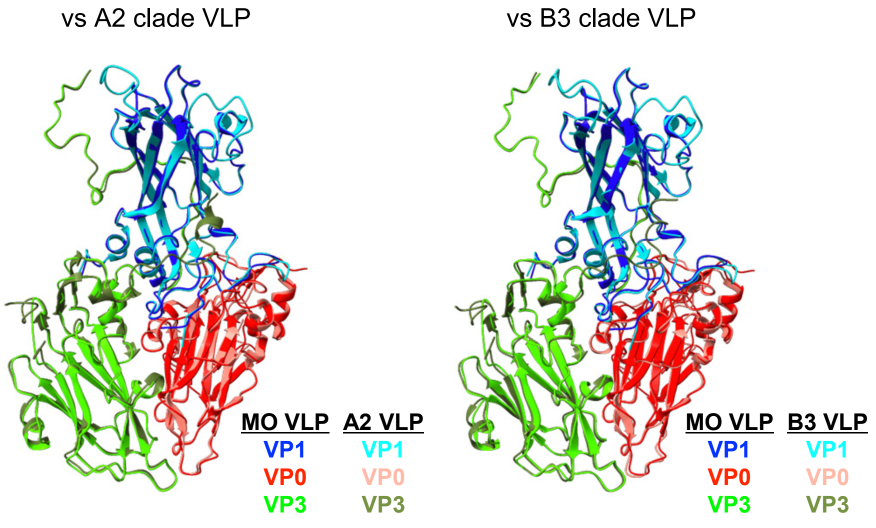

vs A2 clade VLP	Pruned atom pairs	RMSD (Å)
VP1	190	0.459
VP0	186	0.477
VP3	218	0.455

vs B3 clade VLP	Pruned atom pairs	RMSD (Å)
VP1	189	0.438
VP0	187	0.608
VP3	218	0.491

Figure S10. Structural Comparison of VLPs from multiple strains. A structural comparison was performed between the icosahedral asymmetric unit of the VLP from the MO strain (PDB: 9W4I) and those from the A2 subclade (left; PDB: 9C4A) and the B3 subclade (middle; PDB: 9C3J). VP1, VP0, and VP3 subunits of each VLP were superimposed onto the corresponding subunits of the mature virion and the empty particle. Representative structures are shown based on superposition using VP1 as the reference. All structures are displayed as cartoon models. The table in the right panel summarizes the number of pruned atom pairs and root mean square deviation (RMSD) values for each superposition.

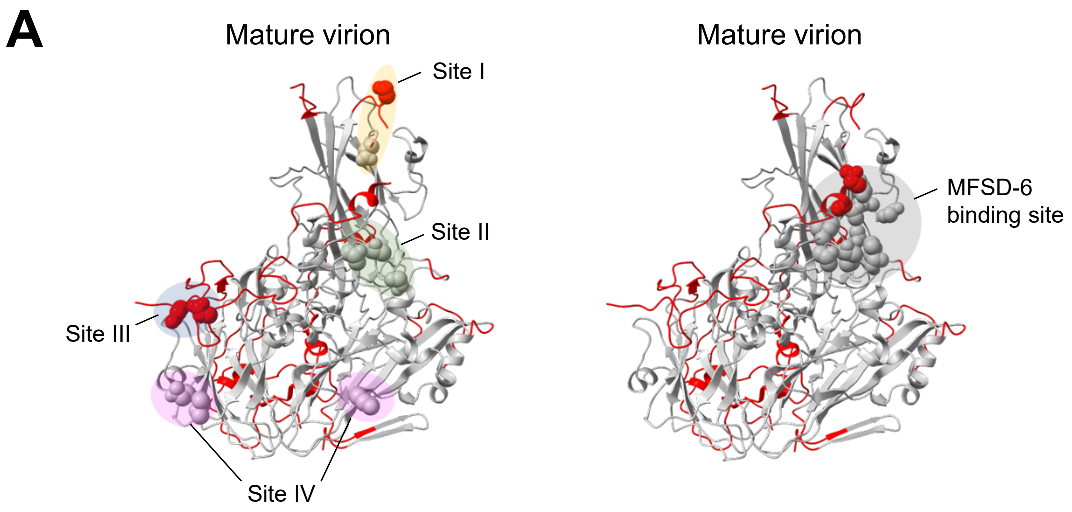

B

VP4 (VP0 in VLP)

Mature virion: MGAQVTRQQTGTHENANIATNGSHITYNQINFYKDSYAASASKQDFSQDPSKFTEPVVEGLKAGAPVLK
VLP: MGAQVTRQQTGTHENANIATNGSHITYNQINFYKDSYAASASKQDFSQDPSKFTEPVVEGLKAGAPVLK

VP2 (VP0 in VLP)

Mature virion: SPSAEACGYSDRVLQLKLGNSAIVTQEAANYCCAYGEWPNYLPDHEAVAIDKPTQPETATDRFYTLKSVK
VLP: SPSAEACGYSDRVLQLKLGNSAIVTQEAANYCCAYGEWPNYLPDHEAVAIDKPTQPETATDRFYTLKSVK

Mature virion: WETGSTGWWWKLPDALNNIGMFGQNVQHHYLYRSGFLIHVQCNATKFHQGALLVVAIPEHQRGAHNTNTS
VLP: WETGSTGWWWKLPDALNNIGMFGQNVQHHYLYRSGFLIHVQCNATKFHQGALLVVAIPEHQRGAHNTNTS

Mature virion: PGFDDIMKGEEGGTFNHPYVLDDGTSLACATIFPHQWINLRTNNSATIVLPWMNAAPMDFPLRHNQWTLA
VLP: PGFDDIMKGEEGGTFNHPYVLDDGTSLACATIFPHQWINLRTNNSATIVLPWMNAAPMDFPLRHNQWTLA

Mature virion: IIPVVPLGTRTTSSMVPITVSIAPMCCEFNGLRHAITQ
VLP: IIPVVPLGTRTTSSMVPITVSIAPMCCEFNGLRHAITQ

VP3

Mature virion: GVPTYLLPGSGQFLTTDDHSSAPALPCFNPTPEMHIPGQVRNMLEVVQVESMMEINNTESAVGMERLKVD
VLP: GVPTYLLPGSGQFLTTDDHSSAPALPCFNPTPEMHIPGQVRNMLEVVQVESMMEINNTESAVGMERLKVD

Mature virion: ISALTDVDQLLFNIPLDIQLDGPLRNTLVGNISRYYTHWSGSLEMTFMFCGSFMAAGKLILCYTPPGGSC
VLP: ISALTDVDQLLFNIPLDIQLDGPLRNTLVGNISRYYTHWSGSLEMTFMFCGSFMAAGKLILCYTPPGGSC

Mature virion: PTTRETAMLGTHIVWDFGLQSSVTLIIPWISGSHYRMFNNDAKSTNANVGYVTCFMQTNLIVPSESSDTC
VLP: PTTRETAMLGTHIVWDFGLQSSVTLIIPWISGSHYRMFNNDAKSTNANVGYVTCFMQTNLIVPSESSDTC

Mature virion: SLIGFIAAKDDFSLRLMRDSPDIGQLDHLHAAEAAYQ
VLP: SLIGFIAAKDDFSLRLMRDSPDIGQLDHLHAAEAAYQ

VP1

Mature virion: IESIIKTATDTVKSEINAELGVVPSLNAVETGATSNTEPEEAIQTRTVINQHGVSETLVENFLSRAALVS
VLP: IESIIKTATDTVKSEINAELGVVPSLNAVETGATSNTEPEEAIQTRTVINQHGVSETLVENFLSRAALVS

Mature virion: KRSFEYKDHTSSTARADKNFFKWTINTRSFVQLRRKLELFTYLRFDAEITILTTVAVNGSGNNTYVGLPD
VLP: KRSFEYKDHTSSTARADKNFFKWTINTRSFVQLRRKLELFTYLRFDAEITILTTVAVNGSGNNTYVGLPD

Mature virion: LTLQAMFVPTGALTPEKQDSFHWQSGSNASVFFKISDPPARITIPFMCINSAYSVFYDGFAGFEKNGLYG
VLP: LTLQAMFVPTGALTPEKQDSFHWQSGSNASVFFKISDPPARITIPFMCINSAYSVFYDGFAGFEKNGLYG

Mature virion: INPADTIGNLCVRIVNEHQPVGFTVTVRVYMKPKHIKAWAPRPPRTLPYMSIANANYKGKERAPNALSAI
VLP: INPADTIGNLCVRIVNEHQPVGFTVTVRVYMKPKHIKAWAPRPPRTLPYMSIANANYKGKERAPNALSAI

IGNRDSVKTMPHNIVNT
IGNRDSVKTMPHNIVNT

Figure S11. Comparison of structurally resolved regions between the mature virion and VLP. (A) Regions that are structurally unresolved in the VLP are highlighted in red on the mature virion structure, displayed as a cartoon model. Antigenic sites are represented as spheres in the left panel and are color-coded according to Figure 4A. MFSD-6 interaction sites are shown as spheres in the right panel. (B) Amino acid sequences of the structural protein subunits (VP4, VP2, VP3, and VP1) were aligned. Structurally resolved regions are shown in black, whereas unresolved (disordered or flexible) regions are depicted in gray. Structural information was obtained from cryo-EM models with the following PDB IDs: 6CRU (mature virion) and 9W4I (VLP).

Table S1. Cryo-EM data collection, refinement, and validation statistics.

	VLP MO strain (EMDB-65634) (PDB 9W4I)
Data collection and processing	
Magnification (Nominal)	81 000
Voltage (kV)	300
Electron exposure (e–/Å^2)	50
Defocus range (μm)	0.11 to 2.59
Pixel size (Å)	1.11
Symmetry imposed	I
Initial particle images (no.)	274 941
Final particle images (no.)	58 404
Map resolution (Å)	2.57
FSC threshold	0.143
Map resolution range (Å)	1.14-3.58
Refinement	
Initial model used	in silico model
Model composition	
Nonhydrogen atoms	4873
B factors (Å^2)	
Protein	84.62
R.m.s. deviations	
Bond lengths (Å)	0.012
Bond angles (°)	1.934
Validation	
MolProbity score	0.58
Clashscore	0.10
Poor rotamers (%)	0.93
Ramachandran plot	
Favored (%)	97.85
Allowed (%)	2.15
Disallowed (%)	0.00
