# Supplementary material for: Comparative immunogenic and structural analysis of virus-like particle and inactivated whole-virion vaccines against enterovirus D68
Source: Mol Ther Nucleic Acids. 2026 May 20;37(2):102957. doi: 10.1016/j.omtn.2026.102957 (PMC13264187; doi:10.1016/j.omtn.2026.102957)
Supplement: Document S2. Article plus supplemental information [file mmc2.pdf]

Molecular Therapy
Nucleic Acids
Original Article

Comparative immunogenic and structural analysis of virus-like particle and inactivated whole-virion vaccines against enterovirus D68

Kota Senpuku,[1,2] Yuta Kunishima,[2,3] Mika Hirose,[4] Tatsuya Karaki,[2,3] Kotaro Taniguchi,[1,2] Chikako Kataoka-Nakamura,[3] Toshiro Hirai,[1,2,5,6] Koubun Yasuda,[7] Etsushi Kuroda,[7] Takayuki Kato,[4,5] Taiki Ito,[2,5,6] and Yasuo Yoshioka[1,2,3,5,6,8,9]

[1]Laboratory of Nano-design for Innovative Drug Development, Graduate School of Pharmaceutical Sciences, The University of Osaka, 1-6 Yamadaoka, Suita, Osaka 565-0871, Japan; [2]Vaccine Creation Group, BIKEN Innovative Vaccine Research Alliance Laboratories, Research Institute for Microbial Diseases, The University of Osaka, 3-1 Yamadaoka, Suita, Osaka 565-0871, Japan; [3]The Research Foundation for Microbial Diseases of Osaka University, 3-1 Yamadaoka, Suita, Osaka 565-0871, Japan; [4]Institute for Protein Research, The University of Osaka, 3-2 Yamadaoka, Suita, Osaka 565-0871, Japan; [5]Center for Advanced Modalities and DDS, The University of Osaka, 3-1 Yamadaoka, Suita, Osaka 565-0871, Japan; [6]Vaccine Creation Group, BIKEN Innovative Vaccine Research Alliance Laboratories, Institute for Open and Transdisciplinary Research Initiatives, The University of Osaka, 3-1 Yamadaoka, Suita, Osaka 565-0871, Japan; [7]Department of Immunology, Hyogo Medical University School of Medicine, 1-1 Mukogawa-cho, Nishinomiya, Hyogo 663-8501, Japan; [8]Global Center for Medical Engineering and Informatics, The University of Osaka, 2-2 Yamadaoka, Suita, Osaka 565-0871, Japan; [9]Center for Infectious Disease Education and Research, The University of Osaka, 1-10 Yamadaoka, Suita, Osaka 565-0871, Japan

Enterovirus D68 (EV-D68) primarily causes respiratory illnesses and has been implicated in acute flaccid myelitis. Although virus-like particle (VLP) and traditional inactivated whole-virion (IWV) vaccines have demonstrated efficacy in mice, their immunological differences remain undetermined. Here, we directly compared the immunogenic and structural properties of VLP and IWV vaccines derived from the same EV-D68 strain under identical conditions. Although VLP induced significantly lower levels of EV-D68-specific IgG than IWV, neutralizing antibody titers and protective effects against viral challenge were comparable between the two groups in mice. Passive transfer experiments in neonatal mice further confirmed protection against lethal infection for both vaccine groups. Notably, in contrast to the IWV vaccine, the VLP vaccine elicited antibodies that preferentially recognized a limited subset of epitopes. Cryo-electron microscopy analyses revealed that VLPs structurally resemble the native virus but display distinct features in regions corresponding to epitopes that show differential antibody reactivity between VLP and IWV vaccines. By integrating structural and immunological analyses, we established a mechanistic framework linking capsid architecture to vaccine-induced antibody specificity. These findings suggest that VLP is a promising EV-D68 vaccine antigen with distinct epitope recognition profiles driven by structural characteristics.

INTRODUCTION

Enterovirus D68 (EV-D68) is a non-polio enterovirus belonging to the genus *Enterovirus* in the family *Picornaviridae*. It is a non-enveloped virus with an icosahedral capsid encasing a single-stranded positive-sense RNA genome.[1–3] Enteroviruses generate multiple types of viral particles during their replication cycle. The P1 polyprotein, translated from the viral RNA genome, is proteolytically processed by the viral 3CD protease into the structural proteins VP0 (a precursor of VP2 and VP4), VP1, and VP3.[1–3] These structural proteins, together with the viral RNA, assemble to form immature virions, during which VP0 is further cleaved into VP2 and VP4, resulting in the production of mature infectious virions. In the absence of encapsidated RNA, the assembly process yields non-infectious empty particles referred to as procapsids.[1–3]

EV-D68 primarily infects the respiratory tract of the pediatric population, causing symptoms such as sore throat, cough, nasal congestion, and fever. Additionally, EV-D68 can induce severe pneumonia, often accompanied by respiratory distress and wheezing.[3,4] Additionally, EV-D68 is associated with acute flaccid myelitis (AFM), a serious poliomyelitis-like neurological condition characterized by the sudden onset of muscle weakness, particularly in the arms or legs, with decreased muscle tone and compromised reflexes.[3–6] EV-D68 mainly affects children, but severe cases have also been observed in adults, especially in the elderly and immunosuppressed individuals.[3–6] Since 2014, there have been repeated outbreaks of EV-D68 worldwide. As the number of EV-D68 cases has increased, the number of AFM cases has shown similar trends.[3–6] Although

Received 10 October 2025; accepted 15 May 2026;
https://doi.org/10.1016/j.omtn.2026.102957.

Correspondence: Yasuo Yoshioka, PhD, Vaccine Creation Group, BIKEN Innovative Vaccine Research Alliance Laboratories, Research Institute for Microbial Diseases, The University of Osaka, 3-1 Yamadaoka, Suita, Osaka 565-0871, Japan.
E-mail: y-yoshioka@biken.osaka-u.ac.jp

© 2026 The Author(s).
Published by Elsevier Inc. on behalf of The American Society of Gene and Cell Therapy.
This is an open access article under the CC BY license (http://creativecommons.org/licenses/by/4.0/).

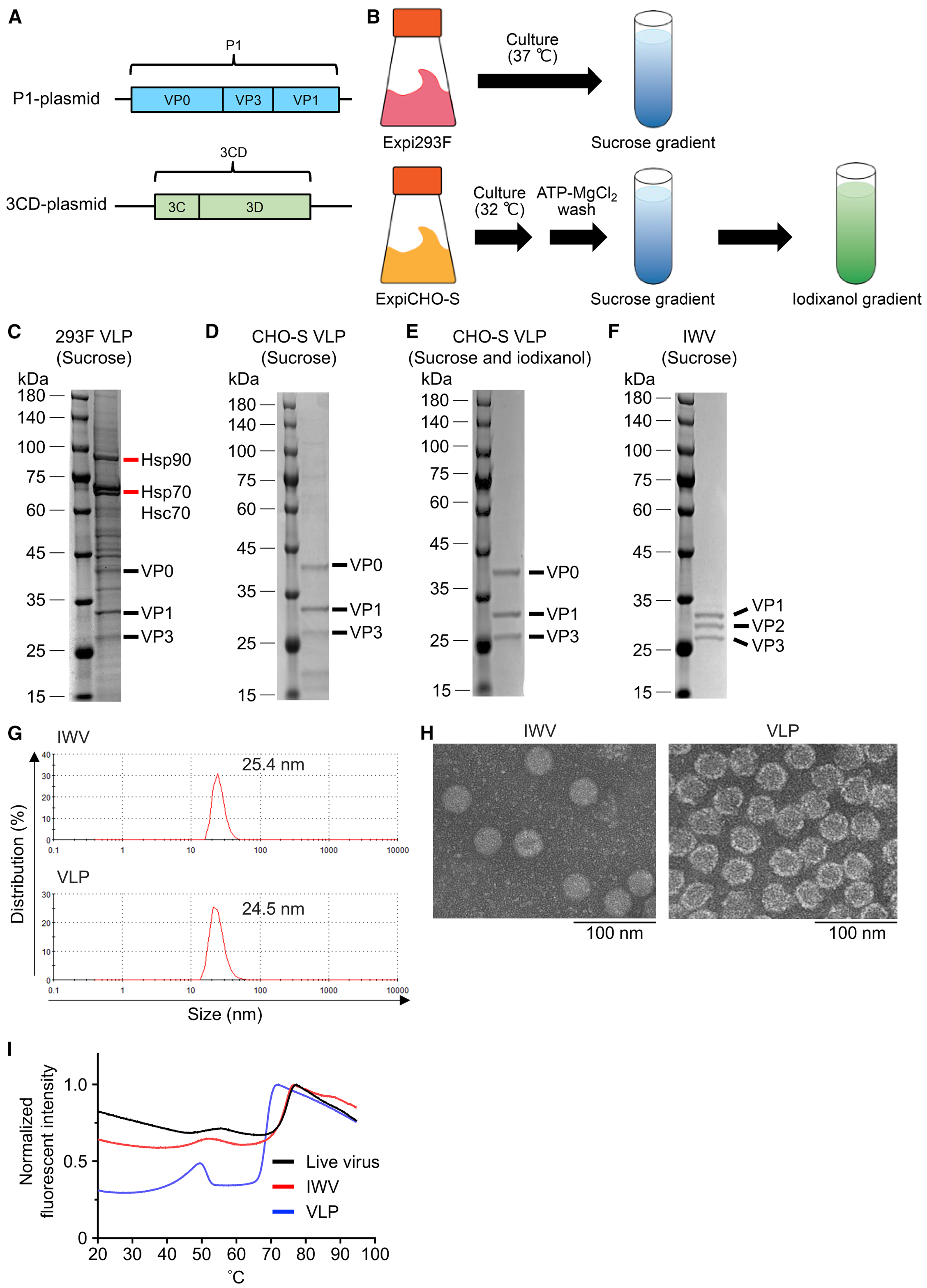

(legend on next page)

EV-D68 continues to pose a threat to public health, there are no approved antiviral therapies or vaccines available. The inactivated whole-virion (IWV) vaccine is a promising candidate against EV-D68, with its use supported by established clinical applications and the demonstrated efficacy of IWV vaccines against poliovirus and EV-A71, both members of the enterovirus genus.[7,8] Previous studies, including ours, have reported that IWV vaccines are effective against EV-D68 in animal models.[9–13] However, EV-D68 exhibits poor replication efficiency in vaccine-producing cell lines, including Vero cells.[11,13,14] Moreover, although Vero-adapted strains can be generated, a risk of decreased immunogenicity through adaptation has been reported.[13]

Virus-like particle (VLP) vaccines have been reported to be effective against a variety of enteroviruses, including EV-D68.[15–18] VLPs are nanoscale structures formed by self-assembly of viral structural proteins.[15–18] Enteroviral VLPs are generally produced by co-expressing P1 and 3CD proteases in mammalian or insect cells.[16,17] Given that the encapsulation of the viral genome and subsequent cleavage of VP0 into VP2 and VP4 do not occur in VLP,[16,17] empty particles and VLP are structurally similar. VLP vaccines have several advantages over IWV vaccines. Because VLP can be produced if the viral genome sequence is available, they can be produced more quickly than live attenuated vaccines or IWV vaccines, which require a lengthy attenuation or adaptation process for vaccine production. Unlike traditional IWV vaccines, VLPs do not require viral propagation in cell culture, thereby avoiding the loss of immunogenicity caused by mutations resulting from attenuation or adaptation. In addition, VLPs do not contain viral genomes nor do they replicate; therefore, they are safe for manufacturing. Recent studies have shown that VLP vaccines against EV-D68 induce the production of neutralizing antibodies and exert protective effects in animal models.[19–22] Additionally, the structure of EV-D68 VLPs has been clarified using cryo-electron microscopy (cryo-EM).[23] However, differences in immunogenicity between VLP and IWV vaccines against EV-D68 have not been fully characterized, and the relationship between vaccine structure and epitope-specific antibody responses remains incompletely understood, particularly regarding the influence of differences in capsid composition and architecture on antigen presentation. Importantly, previous studies have often evaluated VLP and IWV vaccines derived from different viral strains or under nonidentical experimental conditions, hindering the discrimination of platform-specific effects from strain-dependent differences.[19–22]

In the present study, to address these limitations and clarify differences in the immunogenicity between VLP and IWV vaccines against EV-D68, we directly compared the immunogenicity and protective efficacy of VLP and IWV vaccines derived from the same EV-D68 strain in mouse models under identical conditions. Furthermore, we elucidated the three-dimensional structure of VLP using cryo-EM and integrated structural and immunological analyses to investigate the effect of differences in capsid architecture between VLP and IWV vaccines on epitope-specific antibody responses.

RESULTS

Preparation of EV-D68 IWV and VLP

The IWV and VLP were generated from the US/MO/14-18947 strain (MO strain, clade B1) of EV-D68. In IWV particles, VP1, VP2, VP3, and VP4 are present in an approximately 1:1:1:1 stoichiometry, whereas in VLPs, VP0, VP2, and VP3 are present in an approximately 1:1:1 stoichiometry. Furthermore, genome encapsidation and subsequent cleavage of VP0 into VP2 and VP4 do not occur in VLPs. To produce VLP, plasmids encoding the P1 and 3CD proteases were constructed (Figure 1A) and co-transfected into Expi293F cells. Following incubation at 37°C, VLP was harvested and purified by sucrose density gradient ultracentrifugation (Figure 1B, upper). Sodium dodecyl sulfate-polyacrylamide gel electrophoresis (SDS-PAGE) analysis revealed bands corresponding to VP0, VP1, and VP3, consistent with the VLP components (Figure 1C, black lines); however, several contaminating bands were also detected, with notably intense observed bands at ~70 and ~90 kDa (Figure 1C, red lines). Mass spectrometry identified these as heat shock protein 70 (Hsp70), heat shock cognate 71 (Hsc70), and heat shock protein 90 (Hsp90), respectively (Figures 1C and S1). To improve VLP purity, expression was shifted to a lower temperature system (32°C) using ExpiCHO-S cells, and washes with a buffer supplemented with ATP and $MgCl_2$—reported to dissociate Hsps from protein complexes[24,25]—were performed (Figure 1B, lower). This approach substantially reduced the number of contaminating proteins, resulting in clearer VP0, VP1, and VP3 bands (Figure 1D). Further purification by iodixanol density gradient ultracentrifugation yielded highly purified VLP, as confirmed by SDS–PAGE (Figures 1B, lower and 1E). As previously described,[12] IWV was prepared by inactivating the MO strain with β-propiolactone, followed by purification via sucrose density gradient ultracentrifugation (Figure 1F). Quantitative densitometric analysis of Coomassie brilliant blue-stained SDS-PAGE gels further confirmed the purity of IWV and VLP preparations. Sample analysis (1 μg) revealed purities of >90% for both IWV and VLP (Figure S2). Moreover, the amounts of major capsid proteins, including VP1 and VP3, were comparable between the two preparations (Figure S2). These results indicate comparable levels of major structural proteins between the

Figure 1. Preparation and characterization of IWV and VLP

(A) Schematic representation of the construction of plasmids encoding EV-D68 P1 and 3CD. (B) Workflow for the expression and purification of VLP. (C–F) SDS-PAGE analysis of purified IWV and VLP. (C and D) VLP expressed in (C) Expi293F cells and (D) ExpiCHO-S cells purified with sucrose. The Hsp and Hsc identified by mass spectrometry are labeled in red lines. (E) VLP was expressed in ExpiCHO-S cells and further purified using both sucrose and iodixanol (OptiPrep) gradients. (F) IWV was purified by sucrose gradient ultracentrifugation. (G) IWV and VLP particle size distribution was measured by dynamic light scattering. (H) Representative negative-stain TEM images of IWV and VLP. Scale bars, 100 nm. (I) The thermal stability of the non-inactivated virus, IWV, and VLP was assessed using differential scanning fluorimetry (DSF). Data represent the mean of four independent measurements (n = 4) for each sample.

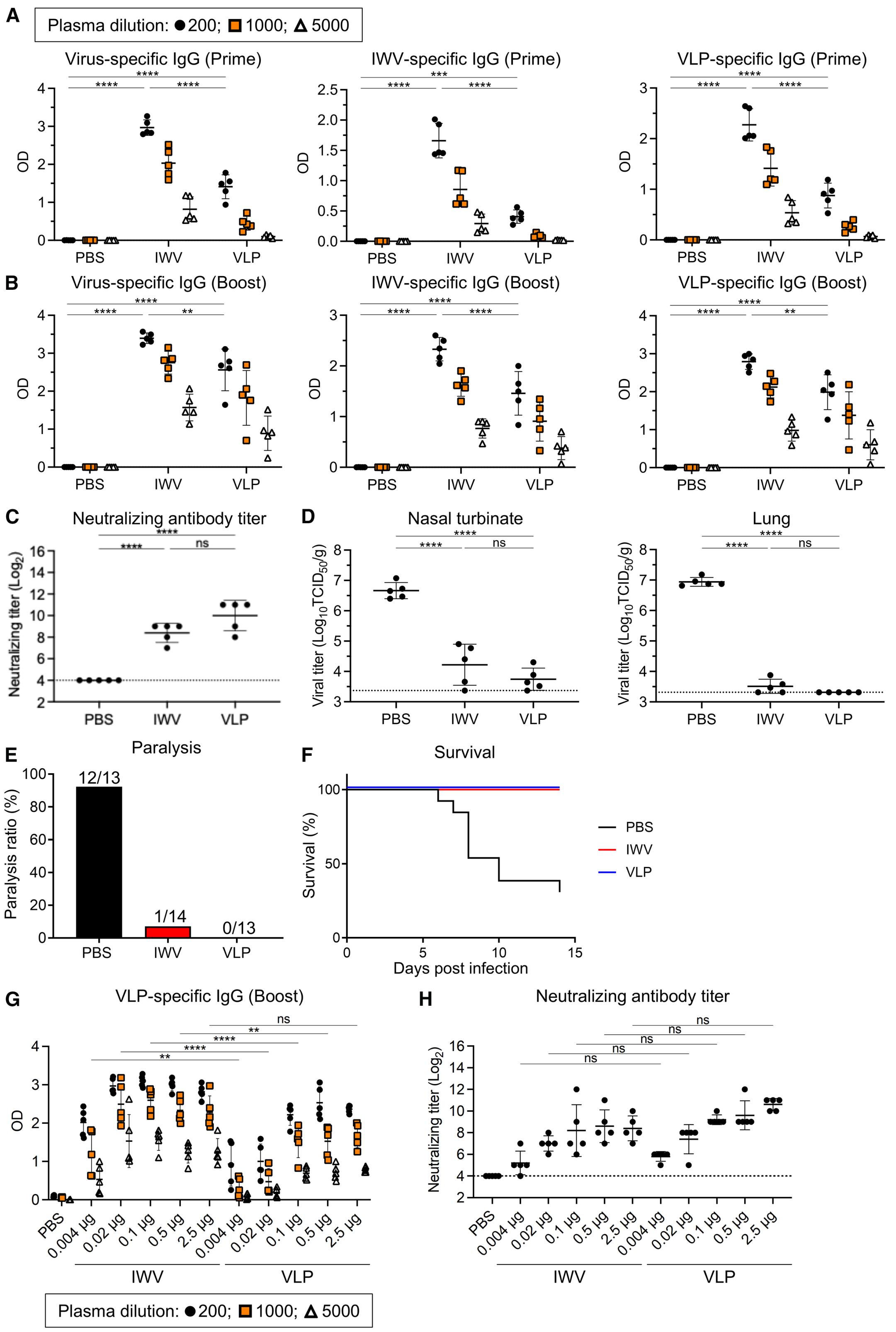

(legend on next page)

two preparations. Western blot analysis using anti-VP1, anti-VP2, and anti-VP3 antibodies confirmed the presence of VP0, VP1, and VP3 in the VLP preparation, whereas VP1, VP2, and VP3 were detected in the IWV (Figure S3). Owing to its small molecular weight (approximately 7.5 kDa) and the lack of a specific antibody, VP4 was not detected by SDS-PAGE (Figure 1F) or western blotting (Figure S3). Dynamic light scattering analysis revealed that both IWV and VLP particles were approximately 25 nm in diameter (Figure 1G), which was consistent with native EV-D68 virions. In addition, negative-stain transmission electron microscopy (TEM) analysis revealed that the IWV and VLP were spherical particles approximately 25 nm in size (Figure 1H). Differential scanning fluorimetry (DSF) was used to compare the thermal stability of viral proteins among IWV, VLP, and non-inactivated viruses. Both the IWV and non-inactivated viruses exhibited elevated signals in the temperature ranges of 50°C–60°C and 70°C–80°C (Figure 1I). These results are consistent with those of previous reports[26] and indicate structural expansion and denaturation. VLP underwent structural expansion and denaturation at lower temperatures than IWV and non-inactivated viruses, indicating that VLP is less thermally stable (Figure 1I).

Comparison of immunogenicity between IWV and VLP

We previously reported that a 0.1 μg IWV vaccine induced robust antibody responses and protective immunity against EV-D68 in mice.[12] The protein concentrations of IWV and VLP were quantified using a BCA assay, and immunizations were performed using 0.1 μg of total protein per dose. Notably, this method did not quantify RNA content in IWV preparations; thus, dosing was standardized based on the total protein content. To directly compare the immunogenicity of the IWV and VLP vaccines, female BALB/c mice were subcutaneously immunized with 0.1 μg of either IWV or VLP on days 0 and 21. Virus-specific IgG levels were assessed by ELISA using non-inactivated virus IWV or VLP as the coating antigen. Regardless of the coating antigen used, the IgG levels were significantly higher in both vaccine groups than in the PBS control group on days 14 and 28 (Figures 2A and 2B). However, the IgG levels in the VLP-immunized group were significantly lower than those in the IWV group (Figures 2A and 2B). Subclass analysis revealed comparable levels of IgG1 and IgG2b between the two vaccine groups, whereas IgG2a levels were significantly lower in the VLP group (Figure S4). Neutralizing antibody titers against the MO strain were significantly elevated in both vaccine groups compared with those in the PBS group (Figure 2C). Notably, in contrast to the total IgG levels, there was no significant difference in neutralizing antibody titers between the IWV and VLP groups (Figure 2C). To determine whether the observed immunogenicity was influenced by the route of administration, we also evaluated immunogenicity following intramuscular immunization. Consistent with the results obtained with subcutaneous immunization, virus-specific IgG levels in the VLP-immunized group were significantly lower, or tended to be lower, than those in the IWV-immunized group after both the prime and booster immunizations (Figure S5A). The VLP vaccine induced neutralizing antibody titers that were comparable to those elicited by the IWV vaccine (Figure S5B). To assess protective efficacy, subcutaneously immunized mice were challenged intranasally with the MO strain, and viral loads in the nasal turbinates and lungs were quantified 12 h post-infection. Both vaccine groups exhibited significantly reduced viral loads compared with the PBS group, with no significant difference observed between the IWV and VLP groups (Figure 2D).

Because the MO strain is not fully adapted to immunocompetent adult mice, we next evaluated the protective capacity of vaccine-induced antibodies using a neonatal mouse model that is more susceptible to EV-D68 infection. Briefly, pooled sera from immunized mice were passively transferred into 1-day-old BALB/c mice, followed by intraperitoneal challenge with the MO strain 1 day later. In the control group, >90% of mice that received sera from PBS-treated mice developed limb paralysis (Figure 2E), and approximately 70% of these mice died within 14 days post-infection (Figure 2F). In contrast, mice that received sera from the IWV- or VLP-immunized groups showed markedly reduced paralysis rates of 7% and 0%, respectively (Figure 2E), and no mortality (Figure 2F). These results suggest that antibodies induced by either vaccine confer strong protection against lethal infection and paralytic disease in a susceptible host.

To further evaluate dose-dependent immunogenicity, mice were immunized with varying doses of IWV or VLP (0.004–2.5 μg) and antibody responses were assessed (Figures 2G and 2H). Although the total IgG levels were consistently lower in the VLP group than in the IWV group (Figure 2G), neutralizing antibody titers remained comparable between the two groups at all doses tested (Figure 2H). Collectively, these results demonstrate that while VLP induces a lower magnitude of virus-specific IgG than IWV, they elicit equivalent levels of neutralizing antibodies and confer comparable protection, supporting the potential of VLP as a promising EV-D68 vaccine antigen.

Figure 2. IWV and VLP immunogenicity

(A–D) Female BALB/c mice were immunized subcutaneously with 0.1 μg of either IWV or VLP. (A and B) Plasma IgG levels specific to whole virus, IWV, or VLP following (A) prime and (B) boost immunization. (C) Neutralizing antibody titers against the MO strain were measured after boost immunization. (D) Viral loads in nasal turbinates and lungs following intranasal challenge with the MO strain. (E and F) Passive transfer protection assay in neonatal mice. Briefly, 1-day-old naive BALB/c mice were intraperitoneally injected with pooled sera collected from immunized mice 7 days after boost immunization, followed by intraperitoneal challenge with the MO strain 1 day later. (E) Limb paralysis and (F) survival rates were monitored every day for 14 days after infection. (G and H) Dose-dependent responses to IWV or VLP vaccines (ranging from 0.004 to 2.5 μg) were immunized subcutaneously. (G) Virus-specific IgG levels in plasma after boost immunization. (H) Neutralizing antibody titers post-boost. (A–D, G, and H) Each group consisted of $n = 5$ mice. (E and F) Each group comprised 13 or 14 mice. (A–D, G, and H) Data are presented as mean ± SD. (C, D, and H) Dotted lines represent the limit of detection. (A, B, and G) Statistical analyses were performed using plasma diluted 1:200 (A and B) or 1:1000 (G). (A–D, G, and H) “ns” indicates not significant. $^{**}p < 0.01$, $^{***}p < 0.001$, and $^{****}p < 0.0001$, as determined by Tukey’s test.

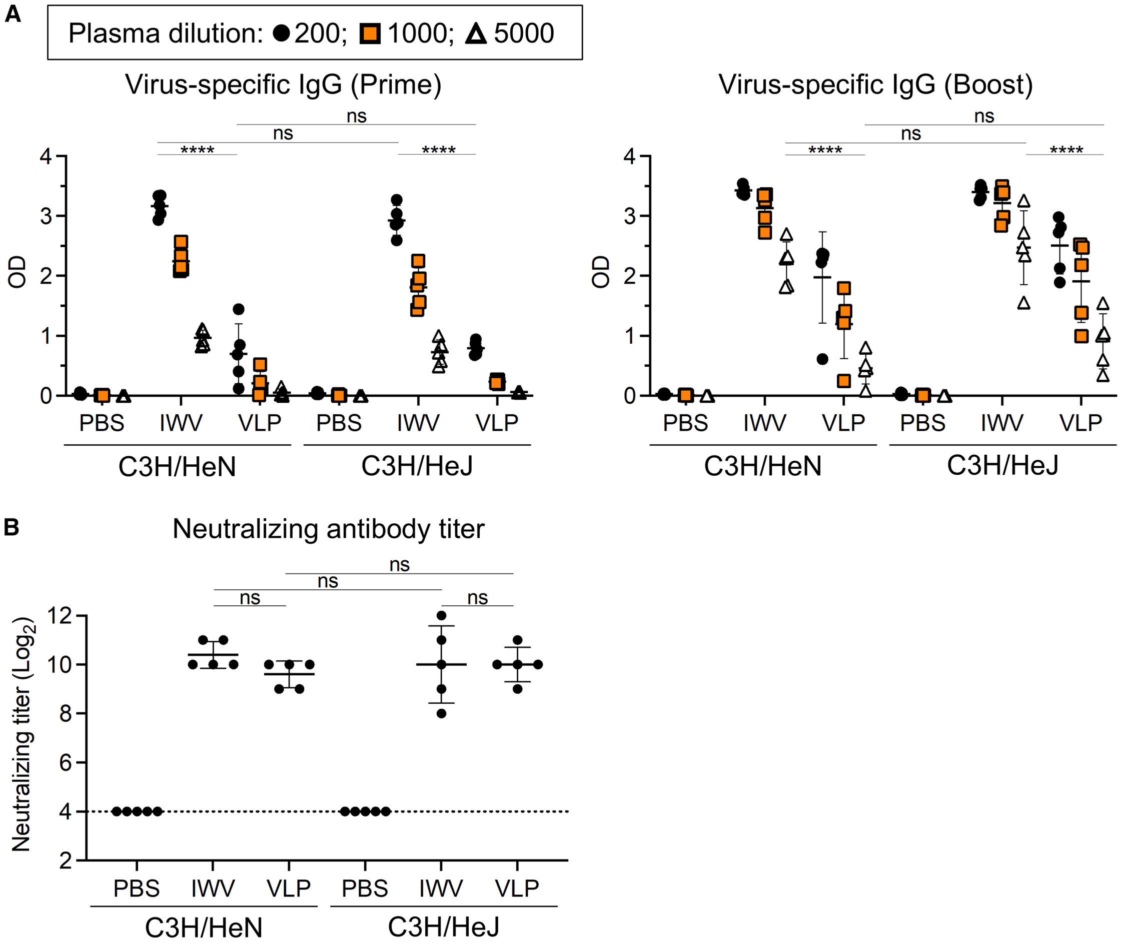

Figure 3. Dispensable role of TLR4 signaling in the vaccine-induced immune response

Female C3H/HeN (wild-type) and C3H/HeJ (TLR4-deficient) mice were immunized subcutaneously with 0.1 μg of IWV or VLP. (A) Virus-specific IgG levels in plasma following prime (left) and boost (right) immunizations. (B) Neutralizing antibody titers measured after boost immunization. $n = 5$ per group. (A, B) Data are presented as mean ± SD. (B) Dotted line represents the limit of detection. Statistical analysis was performed using 200-fold-diluted (Prime) or 5000-fold-diluted (Boost) plasma samples. "ns" denotes not significant. ****$p < 0.0001$, as determined by Tukey's test.

To further evaluate the robustness of the immunogenicity across biological variables, we assessed vaccine responses in male BALB/c mice (Figure S6) and female CB6F1 mice (Figure S7). In both models, the VLP vaccine induced significantly lower EV-D68-specific IgG responses than IWV, whereas neutralizing antibody titers were comparable to those elicited by the IWV vaccine (Figures S6 and S7), consistent with the results obtained in female BALB/c mice. These findings indicated that the immunogenicity of the VLP vaccine is not affected by sex or genetic background, supporting its general applicability.

TLR4- or MyD88-independent immune response

A previous study on a VLP vaccine for EV-A71 reported that innate immune activation via Toll-like receptor 4 (TLR4) signaling, triggered by structural proteins within the VLP, may contribute to vaccine-induced immune responses.[27] To assess the role of TLR4 signaling in the antibody response to IWV and VLP vaccines against EV-D68, we immunized C3H/HeN mice (which express functional TLR4) and C3H/HeJ mice[28] (which possess a TLR4 gene mutation, resulting in defective TLR4 signaling) with IWV or VLP on days 0 and 21, respectively. No significant differences were observed in virus-specific IgG levels (Figure 3A) or neutralizing antibody titers (Figure 3B) between the two mouse strains, regardless of whether they were immunized with IWV or VLP. Consistent with the results obtained in BALB/c mice (Figure 2), VLP immunization induced significantly lower virus-specific IgG levels than IWV immunization (Figure 3A), whereas neutralizing antibody titers remained comparable (Figure 3B). These findings suggest that TLR4 signaling does not contribute to antibody responses induced by IWV or VLP vaccines against EV-D68.

To further explore the involvement of downstream innate immune signaling pathways, we evaluated vaccine-induced antibody responses in MyD88-deficient ($Myd88^{-/-}$) mice. Both male and female

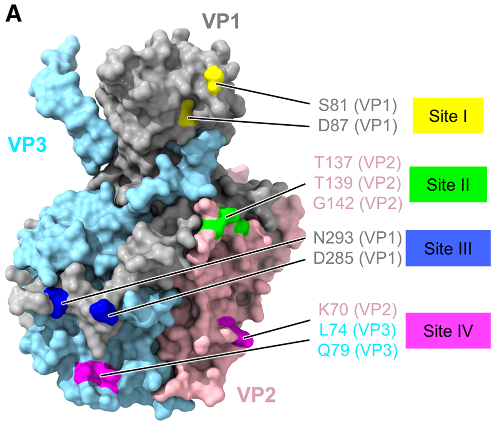

Site	Location	Residues	Peptide	
I	VP1 BC loop	S81 R85 D87	1	YKDHTSSTARADKNFF
II	VP2 EF loop	T137 T139 G142	2	GAHNTNTSPGFD
III	VP1 C-terminus	D285 N293	3	NRDSVKTMPHNIV
IV	VP3 BC loop	L74 Q79	4	SALTDVDQL
	VP2 B *β*-strand	K70	5	LKSVKWETGST

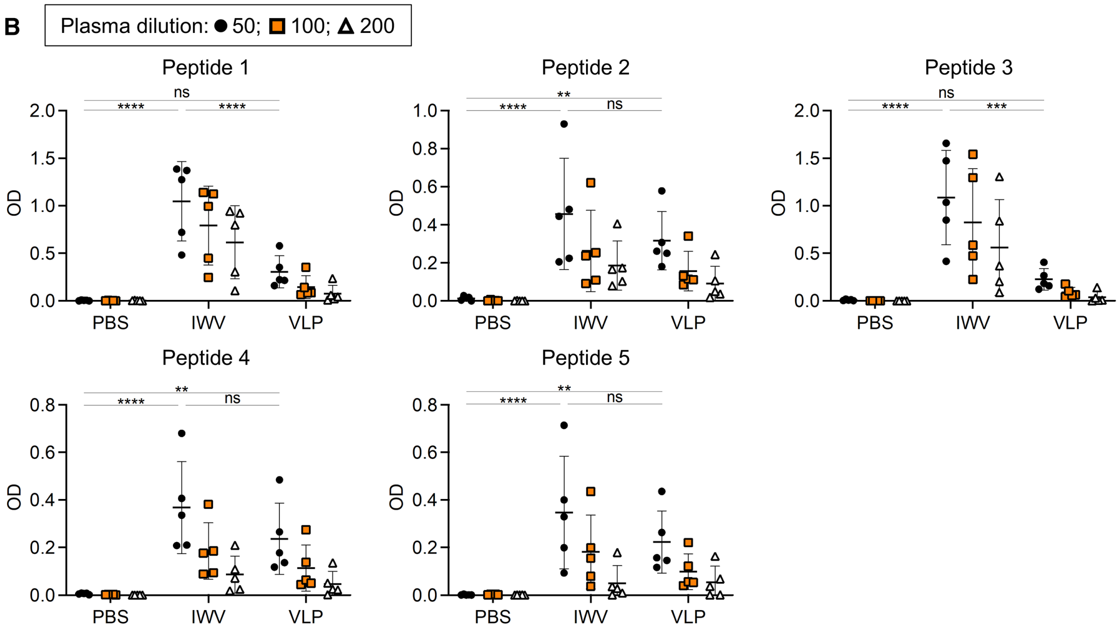

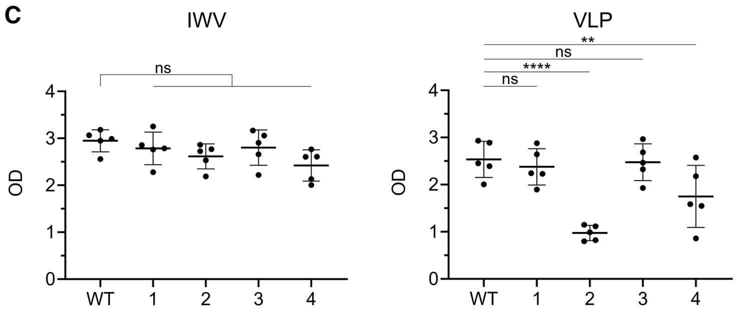

mut VLP	Site and location	Mutation
1	Site I VP1 BC loop	S81L R85Q D87Y
2	Site II VP2 EF loop	T137N T139A G142R
3	Site III VP1 C-terminus	D285G N293D
4	Site IV VP3 BC loop	L74S Q79H
	VP2 B *β*-strand	K70E

Figure 4. Epitope specificities of IgG induced by IWV and VLP vaccines

(A) Localization of neutralizing antigenic sites I–IV and corresponding epitope peptide sequences. Neutralizing antigenic sites I–IV were mapped onto a single icosahedral asymmetric unit of the EV-D68 MO strain capsid, based on the previously reported cryo-EM structure (PDB: 6CSG). VP1, VP2, and VP3 are shown in gray, pink, and cyan, respectively. Neutralizing antigenic sites I, II, III, and IV are highlighted in yellow, green, blue, and magenta, respectively. The accompanying table lists the sequences of epitope peptides spanning the antigenic sites; residues constituting the neutralizing antigenic sites are indicated in red. Molecular graphics were generated using UCSF

(legend continued on next page)

Myd88$^{-/-}$ mice were immunized with IWV or VLP on days 0 and 21. In this model, no significant differences in virus-specific IgG levels were observed between wild-type and *Myd88*$^{-/-}$ mice for either vaccine group (Figure S8). These results suggest that antibody responses induced by IWV and VLP vaccines are largely independent of MyD88-dependent signaling pathways.

Characterization of antibodies induced by IWV and VLP

Recently, four neutralizing antigenic sites (sites I–IV) were identified in the EV-D68 capsid as targets of several monoclonal neutralizing antibodies against EV-D68.[29] These sites and their corresponding amino acid residues are shown in Figure 4A. The epitope peptide sequences surrounding the neutralizing antigenic sites are shown in Figure 4A. Peptide 1 corresponds to site I, peptide 2 corresponds to site II, peptide 3 corresponds to site III, and peptides 4 and 5 correspond to site IV. As described above, we demonstrated that although VLP elicited lower levels of virus-specific IgG than IWV, neutralizing antibody titers were comparable (Figure 2). To investigate the underlying mechanism, we examined whether differences exist in the epitope specificity of virus-specific IgG induced by IWV and VLP vaccination. Specifically, the IgG responses to peptides 1–5 were compared between the two vaccine groups. The IWV group exhibited significantly higher IgG levels against all peptides than the PBS control group (Figure 4B). The VLP group showed IgG responses to peptides 2, 4, and 5 that were comparable to those in the IWV group. However, no significant increase in IgG levels against peptides 1 and 3 was observed in the VLP group compared to the PBS group (Figure 4B). Furthermore, the IgG responses to peptides 1 and 3 in the VLP group were significantly lower than those in the IWV group (Figure 4B). These findings suggest that the epitope recognition pattern of antibodies induced by the VLP vaccine is more limited than that of antibodies generated by the IWV vaccine.

Subsequently, VLPs containing point mutations within the neutralizing antigenic sites (mutVLP1–mutVLP4) were generated, as shown in Figure 4C. The antigen-specific IgG responses induced by the IWV and VLP vaccines against these mutant VLPs were evaluated using the mutant VLPs as coating antigens for ELISA. When post-immunization plasma samples from the IWV group were used, no significant differences in IgG levels were observed between the mutant and wild-type VLPs (Figure 4C, left). In contrast, when post-immunization plasma from the VLP group was used, IgG reactivity to mutVLP1 and mutVLP3 was comparable to that of wild-type VLP, whereas reactivity to mutVLP2 and mutVLP4, particularly mutVLP2, was significantly reduced (Figure 4C, right). These findings demonstrate that IgG elicited by the VLP vaccine predominantly recognizes sites II and IV, while showing minimal binding to sites I and III, in agreement with the results of the previous peptide ELISA. Collectively, these results show that while antibody responses at sites II and IV were comparable between IWV and VLP, the VLP vaccine elicited significantly lower responses at sites I and III.

Cryo-EM structural analysis of VLP

The structures of several EV-D68 strains, including the MO, have been previously reported.[30–32] In a recent study, the structures of VLPs from the B3 and A2 subclades of EV-D68, along with their corresponding IWVs, were resolved.[23] However, the VLP structure of the MO strain used in this study was not determined. To elucidate the potential mechanisms underlying the differing immunogenicity between IWV and VLP vaccines, we determined the structure of the MO strain VLP at 2.57 Å resolution using cryo-EM (Figure S9; Table S1). The structure of the VLP more closely resembled the empty particle or procapsid form rather than the mature virion, with an "opened" particle conformation (Figure 5A). The structure of the MO strain VLP was largely consistent with previously reported EV-D68 VLP structures from other A2 and B3 clade strains (Figure S10).[23] Detailed structural analysis revealed that the pores surrounding the 2- and 3-fold axes were open in the VLP, which is consistent with the empty particle structure (Figure 5B). To confirm this similarity, the capsid proteins VP0, VP1, and VP3 were structurally aligned with their counterparts in empty and mature virions. The root-mean-square deviation (RMSD) values for these subunits, except for VP2, were lower when aligned with the empty particle subunits, indicating that the VLP structure more closely resembled the empty particles even at the subunit level (Figure 5C). This finding supports the conclusion that VLP exhibits greater structural similarity to empty particles than to mature virions (Figure 5C). Notably, compared with the mature virion structure, several regions in the VLP remained unresolved in the cryo-EM VLP structure (Figure S11A). These unresolved regions included antigenic sites I and III, which were well-defined in the mature virion (Figure S11B). These unresolved regions may be structurally flexible or intrinsically disordered VLP segments.

DISCUSSION

In this study, we developed an EV-D68 VLP vaccine using a mammalian cell expression system. Consistent with previous reports,[21,22] VLP expressed in Expi293F cells and purified by sucrose density gradient ultracentrifugation contained substantial amounts of host cell-derived protein contaminants. Our analysis identified these impurities as heat shock proteins, including Hsp70 and Hsp90. Given that these proteins act as molecular chaperones by binding to exposed hydrophobic residues on client proteins,[33] they may interact with hydrophobic residues exposed on the VLP surface. Considering that VLP exhibits lower thermal stability than IWV and may contain structurally unstable regions, it is hypothesized that chaperone binding facilitates proper protein folding at 37°C.

ChimeraX v1.9. (B) Plasma IgG levels specific to epitope peptides following boost immunization with either IWV or VLP. (C) Plasma IgG reactivity to mutant VLPs following boost immunization with IWV or wild-type VLP. Then, 1,000-fold diluted plasma samples were used. Details of the mutations in each mutant are summarized in the table on the right. (B–C) $n = 5$ per group. Data are presented as mean ± SD. (B) Statistical comparisons were performed using 50-fold diluted plasma samples. (B) "ns" indicates not significant. $^{**}p < 0.01$, $^{***}p < 0.001$, and $^{****}p < 0.0001$, as determined by Tukey's test. (C) $^{*}p < 0.05$ and $^{****}p < 0.0001$, as determined by Dunnett's multiple comparison test.

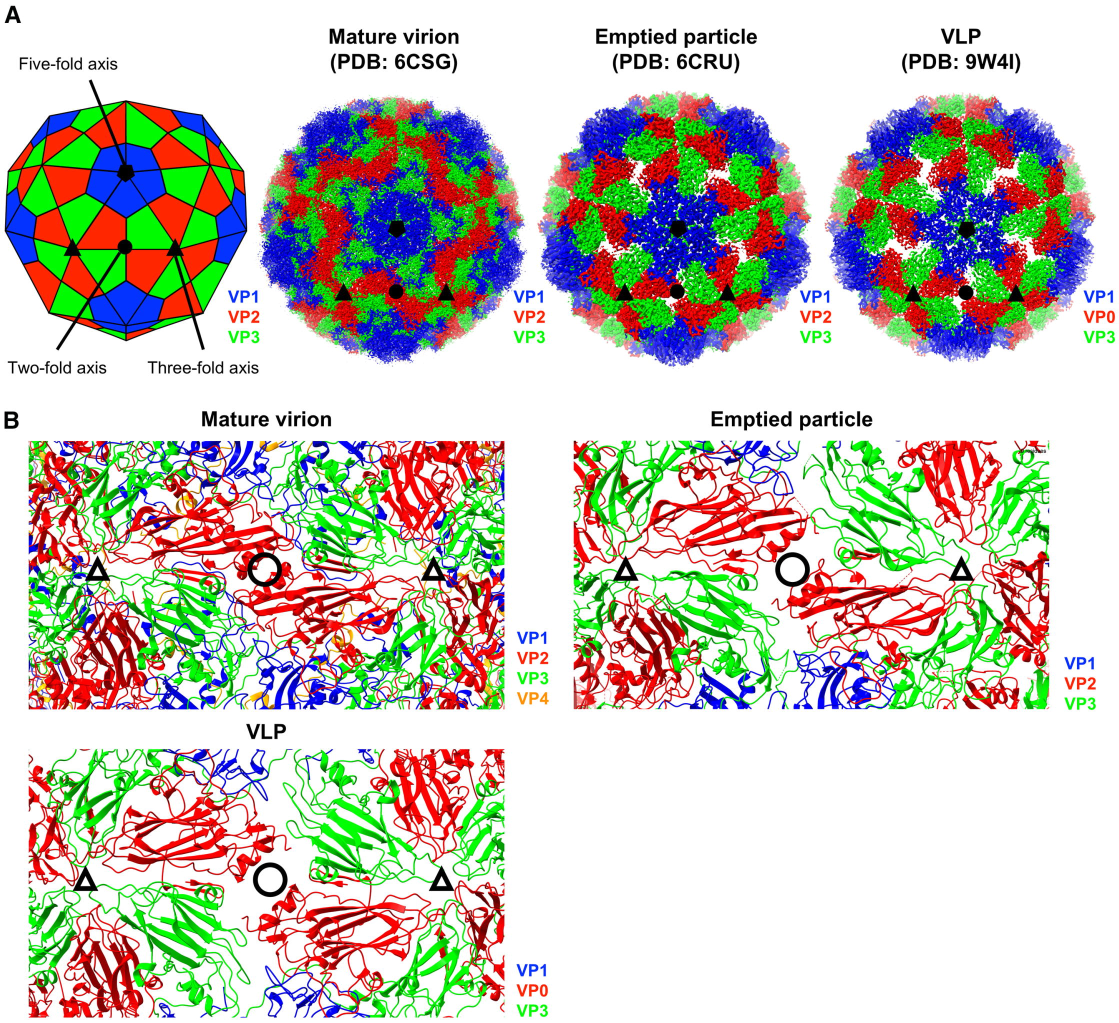

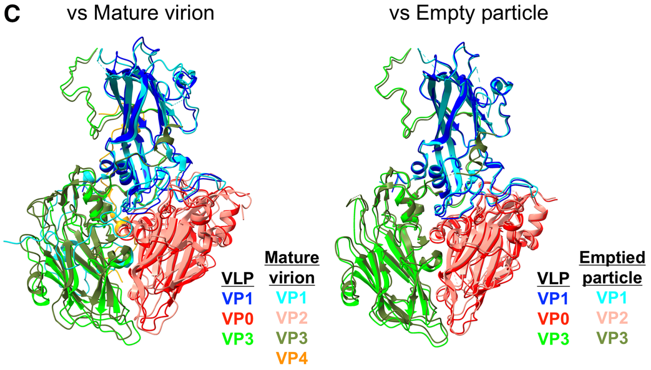

vs Mature virion	Pruned atom pairs	RMSD (Å)
VP1	186	1.028
VP0	199	0.552
VP3	163	0.986
**vs Emptied particle**	**Pruned atom pairs**	**RMSD (Å)**
VP1	189	0.629
VP0	206	0.600
VP3	212	0.832

Figure 5. Cryo-EM structure of EV-D68 MO strain VLP

(A) Cryo-EM density maps showing the overall structures of the mature virion (PDB: 6CSG),[31] empty particle (PDB: 6CRU),[31] and VLP of the EV-D68 MO strain. A schematic illustration of the viral particle is shown in the left-most. The 5-, 3-, and 2-fold symmetry axes are indicated by a pentagon, triangle, and circle, respectively. (B) Ribbon representations of the mature virion (PDB: 6CSG), empty particle (PDB: 6CRU), and VLP structures around the 2-fold axis. The 3-fold and 2-fold axes are denoted by a triangle and circle, respectively. (C) Structural comparison of the icosahedral asymmetric units of the VLP with those of the mature virion (left, PDB: 6CSG) and the empty particle (middle, PDB: 6CRU). VP1, VP0, and VP3 of the VLP were superimposed onto the corresponding subunits of the mature virion and the empty particle. Representative structures are shown based on superposition via VP1. All structures are depicted as ribbon models. The table on the right summarizes the number of pruned atom pairs and root-mean-square deviation (RMSD) values for each superposition.

Accordingly, we optimized the expression and purification protocols, notably by lowering the incubation temperature to 32°C, which is known to enhance correct protein folding compared to 37°C. Additionally, ATP washing steps were performed to dissociate the chaperone proteins. These modifications substantially reduced host protein contamination, which is expected to further improve VLP vaccine production. However, residual host cell-derived components in the final preparations were not fully quantified, and their potential effects on the observed immunogenicity cannot be excluded. This limitation highlights an important area for future investigation.

A previous study compared the abilities of IWV and VLP vaccines to induce neutralizing antibodies and protective immunity against EV-D68[21]; however, different viral strains were used for each, making it unclear whether differences in immune responses arose from vaccine platforms or strain variation. Furthermore, detailed analyses of the antibody epitope specificity are lacking. Importantly, unlike previous studies, we directly compared IWV and VLP vaccines derived from the same EV-D68 strain under identical experimental conditions, enabling direct isolation of vaccine platform-specific effects on antigen structure and epitope-level immune recognition. To address this issue, we produced both vaccines using the same EV-D68 strain and conducted a rigorous immunogenicity comparison. Our findings revealed that neutralizing antibody titers and protective efficacy against viral challenge were comparable between the IWV and VLP vaccines. Consistently, the passive transfer of sera from immunized mice into neonatal mice conferred protection and reduced disease severity in both vaccine groups. In addition, the immunogenicity of both vaccines was not influenced by sex or genetic background, supporting the robustness of the observed humoral immune responses. The robust induction of neutralizing antibodies by the VLP vaccines further supports their potential applicability for vaccine development. Moreover, by integrating structural and immunological analyses, the present study provides insight into the influence of differences in capsid organization on epitope-specific antibody responses, offering a mechanistic perspective that complements previous descriptive studies. However, T cell-mediated immune responses were not evaluated in the present study, which represents an important limitation and a direction for future investigation.

Both the IWV and VLP vaccines elicited strong neutralizing antibody responses, even in the absence of adjuvants. TLR7 signaling, triggered by viral genomic RNA, is known to play a key role in immune activation by IWV vaccines against the influenza virus.[34,35] The immunogenicity of the EV-D68 IWV vaccine is expected to be similarly enhanced by the viral RNA acting as an intrinsic adjuvant via TLR7. In contrast, VLPs do not contain viral RNA; thus, their immunostimulatory capacity cannot be attributed to RNA-mediated TLR7 activation. In addition, studies on VLPs derived from norovirus, a non-enveloped virus similar to EV-D68, have suggested that TLR2 and TLR5 may mediate immune activation via direct interactions with VLPs.[36] We first considered the possibility that VLP-associated structural proteins directly engage innate immune receptors, as proposed in a previous study of EV-A71 VLP vaccines, in which TLR4 signaling contributed to vaccine-induced immune responses.[27] We confirmed that TLR4 did not contribute to immunity induced by EV-D68 VLP. Furthermore, TLR4 signals through MyD88, a central adaptor molecule shared by most TLRs, including TLR2, TLR5, and TLR7. Consistent with the lack of TLR4 involvement, antibody induction by both IWV and VLP vaccines was largely MyD88-independent, suggesting that MyD88-dependent TLR signaling is not the dominant driver of humoral responses in this system. However, the specific innate immune pathways underlying the adjuvant-like effects of these vaccine formulations, including potential contributions from other TLRs or MyD88-independent pathways, remain unclear and warrant further investigation.

We demonstrated distinct differences in epitope-specific IgG responses elicited by the IWV and VLP vaccines. Although both vaccines induced comparable levels of neutralizing antibodies, they differed in the distribution of epitope targets. Antibody responses at sites II and IV were similar between IWV and VLP; however, VLP elicited significantly lower responses at sites I and III. Notably, even in analyses using VLP variants, the VLP preferentially induced antibodies targeting site II. Structural analysis of the MO strain-derived VLP revealed that epitopes corresponding to sites II and IV were well resolved, whereas structural information for sites I and III was lacking, suggesting that these regions may be conformationally flexible or disordered. Therefore, structural flexibility may impair the induction of these site-specific antibodies. Although the neutralizing activities of antibodies directed against sites I–IV have not been fully characterized, those targeting sites I and III may possess lower neutralizing potency and contribute less to overall protective immunity than antibodies against sites II and IV. EV-D68 utilizes sialic acid and heparan sulfate glycosaminoglycans as attachment factors.[29,37,38] Recently, the major facilitator superfamily domain-containing protein 6 (MFSD6) was identified as a functional entry receptor for EV-D68.[39,40] Cryo-EM studies showed that the EV-D68 IL strain (US/IL/14–18952, clade B2), which has high P1 polyprotein amino acid sequence similarity (99.0%) with the MO strain, engages MFSD6 through specific residues in VP1 (K92, T94, G151, L208, T216, N219, and I262), VP2 (T137, T139, and S140), and VP3 (A245 and Q247).[40] Notably, site II corresponded to a region of VP2, which included residues T137, T139, and S140, and was located near the canyon of the viral capsid and overlapped with the MFSD6-binding interface. These findings suggest that antibody targeting site II likely neutralizes the virus by blocking its interaction with MFSD6, highlighting site II as an important target contributing to protective immunity against EV-D68. Although overall virus-specific antibody production was reduced, neutralizing antibody titers were maintained in the VLP group owing to these preserved site II responses. Together, these findings indicate that vaccine platform-dependent differences in capsid architecture selectively shape epitope targeting without compromising overall neutralizing activity.

Cryo-EM showed that the structure of MO strain VLP closely resembled that of VLPs derived from other EV-D68 clades, as reported in a recent study.[23] Notably, similar to VLPs from poliovirus and EV-A71, both characterized by low thermal stability, EV-D68 VLP exhibited inter-capsomeric gaps. These observations suggest that these gaps may contribute to the relatively low thermal stability of the VLP. Combined with the findings of previous studies on improving the thermal stability of picornavirus VLPs,[41,42] the structural insights obtained here may inform the rational design of amino acid substitutions for enhanced thermal stability. Mutations that enhance intersubunit packing, introduce additional hydrogen bonds, or strengthen aromatic interactions at interfacial regions may be particularly effective in stabilizing the capsid, thereby improving the utility of VLP-based EV-D68 vaccines. Furthermore, our data identified site II as a key neutralizing epitope, suggesting that targeted modifications at this site could improve vaccine effects. By integrating cryo-EM structural analysis with epitope-resolved immunological profiling, we provide a mechanistic link between capsid architecture and antibody specificity.

In conclusion, we rigorously compared the immunogenicity of IWV and VLP vaccines against EV-D68 and found that while both conferred comparable protective immunity, the specificity of the antibody responses elicited by each differed. Our findings establish a framework for the rational design of next-generation EV-D68 vaccines and other enteroviral vaccines.

MATERIALS AND METHODS

Ethics

All animal experiments were conducted in accordance with the institutional animal care guidelines of The University of Osaka and were approved by the Animal Care and Use Committee of the Research Institute for Microbial Diseases, The University of Osaka, Japan (protocol number: BIKEN-AP-R01-15-3). Experiments using MyD88-deficient mice were performed in accordance with the guidelines for the care and use of animals approved by Hyogo Medical University (protocol numbers 23-018AGP and 26-015AGP). The use of EV-D68 was reviewed and approved by the Institutional Review Board of the Research Institute for Microbial Diseases, The University of Osaka (protocol number: BIKEN-00184-004).

Cell culture

Expi293F cells (Thermo Fisher Scientific, Waltham, MA, USA) were maintained in Expi293 expression medium (Thermo Fisher Scientific) under conditions of 8% CO_2 and 37°C using a rotary shaking incubator. ExpiCHO-S cells (Thermo Fisher Scientific) were cultured in ExpiCHO expression medium under the same environmental conditions. RD-A cells were kindly provided by Dr. Hiroyuki Shimizu (National Institute of Infectious Diseases, Tokyo, Japan) and cultured in high-glucose Dulbecco's modified Eagle's medium (DMEM) supplemented with 10% fetal bovine serum (FBS), 1% penicillin, and 1% streptomycin. All cultures were maintained at 37°C in a humidified incubator with 5% CO_2.

Viruses

The EV-D68 US/MO/14–18947 strain (MO strain, clade B1) was obtained from the American Type Culture Collection (ATCC, Manassas, VA, USA) and stored at −80°C until use. Viral titers were determined using the Karber method and expressed as the 50% tissue culture infectious dose ($TCID_{50}$).

Mice

Six-week-old female and male BALB/c mice and female CB6F1 mice were procured from Japan SLC (Shizuoka, Japan), whereas female C3H/HeN and C3H/HeJ mice were procured from CLEA Japan (Tokyo, Japan). MyD88-deficient BALB/c mice were bred and maintained at Hyogo Medical University School of Medicine, and animals aged 13–14 weeks were used for the experiments.[43] Age-matched wild-type BALB/c mice were used as controls. All animals were housed under a 12 h light/dark cycle with free access to animal feed and water. The mice were anesthetized via intraperitoneal injection of a combination of three agents: 0.3 mg/kg medetomidine (Nippon Zenyaku Kogyo Co., Ltd., Tokyo, Japan) for sedation, analgesia, and muscle relaxation; 4 mg/kg midazolam (Maruishi Pharmaceutical Co., Ltd., Osaka, Japan) for sedation; and 5 mg/kg butorphanol (Meiji Animal Health Co., Ltd., Kumamoto, Japan) for analgesia.

VLPs

Codon usage of the P1 and 3CD genes from the EV-D68 MO strain (GenBank accession number KM851225.1) was optimized for expression in mammalian cells. In addition, a series of P1 gene variants containing mutations in the neutralizing antigenic sites I–IV, as previously identified,[29] were constructed to generate mutant VLPs. The site I mutant included substitutions in VP1 (S81L, R85Q, and D87Y), the site II mutant in VP2 (T137N, T139A, and G142R), the site III mutant in VP1 (D285G and N293D), and the site IV mutant in VP2 (K70E) and VP3 (L74S and Q79H). The corresponding DNA fragments were cloned into the pcDNA3.4 expression vector (Invitrogen, Waltham, MA, USA). VLPs were produced using either Expi293 or ExpiCHO expression systems (Thermo Fisher Scientific), according to the manufacturer's protocols.

VLP production by Expi293F cells

Expi293F cells were seeded at 3×10^6 cells/mL in Expi293 expression medium and transfected with P1 and 3CD plasmids using ExpiFectamine 293 according to the manufacturer's recommendations. Following an 18–22 h incubation at 37°C with 8% CO_2 on a rotary shaker (120 rpm), transfection enhancers 1 and 2 were added. Cultures were maintained for 4 days, then harvested and clarified via sequential centrifugation (300 × *g* for 5 min, 2,500 × *g* for 20 min [two times], and 16,000 × *g* for 45 min) at 4°C. The supernatant was filtered (0.2 μm), and VLP was pelleted by ultracentrifugation through a 20% sucrose cushion (141,000 × *g*, 3 h, 4°C) followed by a 10%–40% sucrose gradient (130,000 × *g*, 4 h, 4°C). VLP-containing fractions were identified by VP1 western blotting (catalog number: GTX132313; dilution: 1/3,000; GeneTex, Irvine, CA, USA), concentrated using a 100 kDa cutoff filter, and resuspended in PBS.

VLP production by ExpiCHO-S cells

For ExpiCHO-S expression, cells were adjusted to 6×10^6 cells/mL and transfected with P1 and 3CD plasmids using ExpiFectamine CHO. After being maintained for 18–22 h at 37°C and 8% CO_2, enhancer and feed were added. Cultures were incubated for 4 days at 32°C and 5% CO_2. Harvesting and clarification were performed following the Expi293F protocol. VLP was isolated via 20% sucrose cushion ultracentrifugation, extracted into ATP-$MgCl_2$ buffer (pH 7: Tris-HCl 50 mM, NaCl 1 M, Triton X-100 0.5%, ATP 10 mM, and $MgCl_2$ 20 mM), incubated 1 h at 4°C, clarified, then subjected to 10%–40% sucrose density gradient (130,000 × *g*, 4 h, 4°C), and further purified using a 10%–50% iodixanol (OptiPrep) gradient (120,000 × *g*, 16 h, 4°C). The fractions containing VLP were pooled, concentrated, and suspended in PBS. The protein concentration was measured using a BCA Protein Assay kit (Thermo Fisher Scientific).

IWV production

RD-A cells at ~80% confluency in DMEM + 10% FBS were switched to VP-SFM (1×) (ThermoFisher Scientific), supplemented with 4 mM L-alanyl-l-glutamine (Nacalai Tesque, Kyoto, Japan), 1% penicillin, and 1% streptomycin (WAKO, Osaka, Japan), and infected with EV-D68 at 33°C. When the full cytopathic effect was observed (2–3 days), cultures were harvested, clarified by centrifugation (2,100 × *g*, 10 min) at 4°C, and filtered (0.2 μm). Virions were inactivated with 0.05% β-propiolactone at 4°C for 24 h, followed by hydrolysis at 37°C for 2 h. Virions were purified via ultracentrifugation through a 20% sucrose cushion (141,000 × *g*, 3 h) and a 10%–40% sucrose gradient (130,000 × *g*, 4 h), and inactivated virion fractions (identified via VP2 western blotting) were concentrated (100 kDa cutoff) and resuspended in PBS. Protein content was assessed by BCA.

SDS-PAGE and western blotting

Samples were mixed with reducing sample buffer, heated at 95°C for 5 min, and loaded onto 10% SDS-PAGE gels (Nacalai Tesque). The gels were then stained with EzStain Aqua (ATTO, Tokyo, Japan). Proteins were transferred to PVDF membranes, blocked with 5% skim milk in PBS containing 0.05% Tween 20 (PBS-T), and probed with rabbit polyclonal anti-EV-D68 VP1 (catalog number: GTX132313, dilution: 1:3,000, GeneTex), anti-EV-D68 VP2 (catalog number: GTX132314, dilution: 1:5,000, GeneTex), or anti-EV-D68 VP3 (catalog number: GTX132315, dilution: 1:3,000, GeneTex). After three additional washes with PBS-T, the membranes were exposed to horseradish peroxidase (HRP)-conjugated anti-rabbit IgG antibody (catalog number: 458; dilution: 1:5,000; Medical and Biological Laboratories, Tokyo, Japan). Protein signals were visualized using the ChemiDoc Touch Imaging System (Bio-Rad).

Size measurement

The IWV and VLP sizes were analyzed with the Zetasizer Nano-ZS (Malvern Panalytical Ltd., Worcestershire, UK).

Negative-stain TEM

The hydrophilic carbon-formvar grid was soaked in 20 mL of the sample solution for 10 min. Subsequently, cells were washed with distilled water, stained with 2% uranyl acetate, and dried on a filter paper to remove excess moisture. After natural drying, the samples were observed using an 80 kV JEOL JEM-1400pLus electron microscope. Images were captured using an Olympus Veleta 2 K × 2 K side-mounted TEM charge-coupled device camera.

DSF

The thermal stability of the protein samples was evaluated by DSF using SYPRO Orange dye (Thermo Fisher Scientific). Each reaction (15 μL total volume) contained 1 μg of protein diluted in PBS and SYPRO orange at a final concentration of 9×, which was prepared by diluting the 5,000× stock solution in dimethyl sulfoxide. Reactions were assembled in a 384-well PCR plate (LightCycler 480 Multiwell Plate 384, white, Roche, Basel, Switzerland) and sealed with optical adhesive film to prevent evaporation. Fluorescence measurements were carried out using a real-time PCR instrument (LightCycler 480 II, Roche). Samples were subjected to a temperature gradient from 20°C to 95°C at a ramp rate of 3.6°C/min, with fluorescence recorded ten times per 1°C increment. Each sample was analyzed in quadruplicate.

Immunization

IWV or VLP preparations in 50 μL PBS were injected subcutaneously into the tail base or intramuscularly into the tibialis anterior muscle on days 0 and 21. The antigen dose was calculated based on the protein content measured using the BCA assay. Blood was drawn on days 14 and 28, and the plasma was stored at −30°C. Plasma samples were heat-inactivated at 56°C for 30 min before use in neutralization assays.

Detection of antigen-specific IgG antibodies

Antigen-specific IgG antibodies were detected using ELISA. First, the antigens were coated on a 96-well half-area flat-bottom plate (Corning, NY, USA) at a concentration of 0.6 μg/mL in carbonate buffer. The antigens used were non-inactivated virus, IWV, wild-type VLP, and mutant VLPs. Next, 1% Block Ace (DS Pharma Biomedical, Osaka, Japan) was added and incubated for 1 h at room temperature for blocking. Next, the samples were serially diluted with 0.4% Block Ace, and the diluted samples were added. After incubation at room temperature for 2 h, HRP-conjugated goat anti-mouse IgG (catalog number: 1030-05, dilution: 1/5,000, SouthernBiotech, Birmingham, AL, USA), IgG1 (catalog number: 1070-05, dilution: 1/5,000, SouthernBiotech), IgG2a (catalog number: 1083–05, dilution: 1/5,000, SouthernBiotech), and IgG2b (catalog number: 1093-05, dilution: 1/5,000, SouthernBiotech) and incubated at room temperature for 1 h. The color reaction was performed using tetramethylbenzidine and terminated using H_2SO_4. Optical density 450–570 ($OD_{450–570}$) was determined using a microplate reader (PowerWave HT; Bio-Tek Instruments, Inc., Winooski, VT, USA).

Detection of IgG antibodies binding to epitope peptides

IgG antibodies bound to epitope peptides were detected using ELISA with a peptide-coating kit (Takara Bio Inc., Shiga, Japan). Each peptide was coated at 80 μg/mL on a reaction plate using the reaction

buffer and coupling reagent included in the kit. Blocking solution was added and incubated at room temperature for 1 h. Plasma samples were diluted in 0.4% Block Ace and incubated for 2 h at room temperature. HRP-conjugated goat anti-mouse IgG was added, and the cells were incubated at room temperature for 1 h. The color reaction was performed using tetramethylbenzidine and stopped using 2 N H_2SO_4. $OD_{450–570}$ was measured using a microplate reader. The peptide sequences are as follows: peptide 1, YKDHTSSTARADKNFF; peptide 2, GAHNTNTSPGFD; peptide 3, NRDSVKTMPHNIV; peptide 4, SALTDVDQL; and peptide 5, LKSVKWETGST. The peptides were synthesized using GenScript (Piscataway, NJ, US).

Neutralization assay

Heat-inactivated plasma samples were serially diluted 2-fold in DMEM containing 2% FBS in 96-well plates. Diluted plasma samples (50 μL/well) were incubated with 100 $TCID_{50}$ of EV-D68 in DMEM containing 2% FBS (50 μL/well) for 1 h at 37°C. Subsequently, 1.5×10^4 RD-A cells suspended in DMEM containing 10% FBS were added to each well (100 μL/well), and the plates were incubated for 7 days in 5% CO_2 at 33°C.[44] The highest plasma dilution at which cytopathic effects (CPEs) were completely inhibited was defined as the neutralizing antibody titer.

EV-D68 challenge

Ten days after booster immunization, the mice were anesthetized and intranasally challenged with 5.0×10^6 $TCID_{50}$ of EV-D68 in 20 μL PBS (10 μL in each nostril). At 12 h post-challenge, nasal turbinates and lungs were harvested and placed in a screw-capped tube containing 4.0 mm stainless steel beads (TAITEC, Saitama, Japan). A volume of 500 μL of DMEM was added to each sample, and the tissue samples were lysed with the μT-12 bead crusher (TAITEC). The samples underwent centrifugation at $10{,}000 \times g$ for 5 min at 4°C. The viral load in the supernatant was measured.

Passive transfer experiments

Naive 1-day-old BALB/c mice were intraperitoneally administered 50 μL of heat-inactivated sera pooled from adult mice that had been immunized twice with IWV or VLP. One day later, neonatal mice were challenged by intraperitoneal injection with 3.2×10^6 $TCID_{50}$ of EV-D68 (100 μL). Survival and development of paralysis were monitored daily for 14 days post-infection.

Cryo-EM imaging and structural determination

Grids (Quantifoil Au R1.2/1.3 300 mesh; SPT Labtech, Melbourn, UK) were glow-discharged for 30 s to hydrophilize the surface. The VLP sample solution (3 μL) was applied to a grid at 4°C under 100% humidity. After blotting the excess solution on the grid with a filter paper, the grids were rapidly vitrified in liquid ethane using a Vitrobot Mark IV (Thermo Fisher Scientific) for cryo-EM image acquisition. Cryo-EM data were collected using a Titan Krios (Thermo Fisher Scientific) equipped with a Cs corrector (CEOS GmbH, Heidelberg, Germany) operating at 300 keV in the EFTEM nanoprobe mode. Images were acquired as movies using a BioQuantum energy filter (slit width of 20 eV) and a K3 camera (Gatan, Inc., Pleasanton, CA, USA) in the electron counting mode. A total of 819 movies were collected at a dose rate of 7.82 e^-/pixel/s, pixel size of 0.87 $Å^2$, and total dose of 50 $e^-/Å^2$. Automated data collection was performed using SerialEM software[45] with a nominal defocus range of −0.6 to −1.8 μm. All image processing was carried out using cryoSPARC version 4.6.0 software.[46] Motion correction and contrast transfer function (CTF) estimation were performed. An initial set of 37 particles was manually picked and subjected to 2D classification. Representative class averages from this step were used as templates for automated particle picking. Using the template-based particle picking approach, 61,454 particles were selected. These particles were subjected to ab initio reconstruction into five classes. Particles belonging to the two best-resolved classes (totaling 60,620 particles) were selected and further classified using heterogeneous refinement into two classes. Particles from the better-resolved class (58,415 particles) were refined using homogeneous refinement, followed by local motion correction. The resulting corrected particles were then subjected to non-uniform refinement to obtain the final reconstruction. After motion correction of movies and contrast transfer function parameter estimation, 37 particles were manually picked and 2D classification was performed. Better averaged images were used as a template for template picking, after which 61,454 particles were picked. A flowchart of the image processing procedure is shown in Figure S9. The overall resolution of the MO strain VLP density map was 2.57 Å. The initial model was generated using AlphaFold3, and rigid-body fitting was performed in ChimeraX. Model refinement was carried out using ISOLDE version 1.6,[47] and residues that were not supported by visible density were removed from the final model. Detailed statistics are provided in Table S1. Structural visualization and analysis were performed with ChimeraX 1.9,[48] and structural superposition was performed using Matchmaker in ChimeraX.

Statistical analysis

Statistical analysis was performed using Prism 10 software version 10.4.0 (GraphPad software, San Diego, CA, USA). Data are expressed as mean and standard deviation (SD). Tukey's test, Student's *t* test, or Dunnett's multiple comparison test was used for significance testing. Statistical significance was set at $p < 0.05$. Each experiment was performed at least twice.

DATA AND CODE AVAILABILITY

The findings of this study are supported by the data reported in the article and the supplementary materials. The cryo-EM map of the EV-D68 MO strain VLP has been deposited in the Electron Microscopy DataBank with accession code EMD-65634. Atomic coordinates were deposited in the Protein DataBank (PDB) under the accession code PDB: 9W4I. Please contact the lead researcher Yasuo Yoshioka (y-yoshioka@biken.osaka-u.ac.jp) for additional information or requests concerning resources and reagents.

ACKNOWLEDGMENTS

This study was supported by grants from the Japan Society for the Promotion of Science, Japan (JSPS KAKENHI grant nos. JP23K27343 and JP24K22020 to Y.Y.), Japan Agency for Medical Research and Development, Japan (AMED grant nos. JP223fa627002 to Y.Y. and JP223fa727001 to E.K.), the All-Osaka U Research in "The Nippon Foundation-Osaka University Project for Infectious Disease Project" (to Y.Y.), the Japan Science

and Technology Agency, Japan (JST SPRING grant no. JPMJSP2138 to K.S.), and The Research Foundation for Microbial Diseases of Osaka University (BIKEN), Japan. We acknowledge Hiroko Omori and Akinori Ninomiya (Core Instrumentation Facility, Research Institute for Microbial Diseases, The University of Osaka, Japan) for their support with negative-stain TEM assessment and mass spectrometry.

AUTHOR CONTRIBUTIONS

K.S. and Y.Y. designed the experiments. K.S., Y.K., M.H., T. Karaki., K.T., K.Y., E.K., T. Kato, and T.I. performed the experiments and analyzed and interpreted the data. K.S., Y.K., M.H., C.K.-N., T.H., T. Kato, and T.I. contributed to the experimental design and edited the manuscript. K.S., Y.K., T.I., and Y.Y. drafted the manuscript. Y.Y. supervised the study. All authors have read and agreed to the final version of the manuscript for publication.

DECLARATION OF INTERESTS

Y.K., T. Karaki, C.K.-N., and Y.Y. were employed by the Research Foundation for Microbial Diseases at Osaka University.

SUPPLEMENTAL INFORMATION

Supplemental information can be found online at https://doi.org/10.1016/j.omtn.2026.102957.

REFERENCES

1. Baggen, J., Thibaut, H.J., Strating, J.R.P.M., and van Kuppeveld, F.J.M. (2018). The life cycle of non-polio enteroviruses and how to target it. Nat. Rev. Microbiol. *16*, 368–381. https://doi.org/10.1038/s41579-018-0005-4.

2. Elrick, M.J., Pekosz, A., and Duggal, P. (2021). Enterovirus D68 molecular and cellular biology and pathogenesis. J. Biol. Chem. *296*, 100317. https://doi.org/10.1016/j.jbc.2021.100317.

3. Grizer, C.S., Messacar, K., and Mattapallil, J.J. (2024). Enterovirus-D68 - A Reemerging Non-Polio Enterovirus that Causes Severe Respiratory and Neurological Disease in Children. Front. Virol. *4*, 1328457. https://doi.org/10.3389/fviro.2024.1328457.

4. Clopper, B.R., Lopez, A.S., Goldstein, L.A., Ng, T.F.F., Toepfer, A.P., Staat, M.A., Schlaudecker, E.P., Sahni, L.C., Boom, J.A., Schuster, J.E., et al. (2025). Enterovirus D68-Associated Respiratory Illness in Children. JAMA Netw. Open *8*, e259131. https://doi.org/10.1001/jamanetworkopen.2025.9131.

5. Larsson, S.B., Vracar, D., Karlsson, M., Ringlander, J., and Norder, H. (2022). Epidemiology and clinical manifestations of different enterovirus and rhinovirus types show that EV-D68 may still have an impact on severity of respiratory infections. J. Med. Virol. *94*, 3829–3839. https://doi.org/10.1002/jmv.27767.

6. Jorgensen, D., Grassly, N.C., and Pons-Salort, M. (2025). Global age-stratified seroprevalence of enterovirus D68: a systematic literature review. Lancet Microbe *6*, 100938. https://doi.org/10.1016/j.lanmic.2024.07.001.

7. Bandyopadhyay, A.S., Lopez Cavestany, R., Blake, I.M., Macklin, G., Cooper, L., Grassly, N., Nery, A.L.M.D.S., and Mach, O. (2024). Use of inactivated poliovirus vaccine for poliovirus outbreak response. Lancet Infect. Dis. *24*, e328–e342. https://doi.org/10.1016/S1473-3099(23)00505-4.

8. Yan, X., Liu, Y., Chen, F., Chang, Z., Zhang, Z., Pons-Salort, M., and Grassly, N.C. (2025). The efficacy and effectiveness of enterovirus A71 vaccines against hand, foot, and mouth disease: A systematic review and meta-analysis. PLoS One *20*, e0323782. https://doi.org/10.1371/journal.pone.0323782.

9. Zhang, C., Zhang, X., Dai, W., Liu, Q., Xiong, P., Wang, S., Geng, L., Gong, S., and Huang, Z. (2018). A Mouse Model of Enterovirus D68 Infection for Assessment of the Efficacy of Inactivated Vaccine. Viruses *10*, 58. https://doi.org/10.3390/v10020058.

10. Zheng, H., Yang, Z., Li, B., Li, H., Guo, L., Song, J., Hou, D., Li, N., Yang, J., Wu, Q., et al. (2020). Single B cells reveal the antibody responses of rhesus macaques immunized with an inactivated enterovirus D68 vaccine. Arch. Virol. *165*, 1777–1789. https://doi.org/10.1007/s00705-020-04676-6.

11. Raychoudhuri, A., Naru, A.K., Kanubothula, S.R., and Uddala, R. (2021). Development of an experimental inactivated vaccine from Vero cell adapted Enterovirus D68. Virus Res. *304*, 198528. https://doi.org/10.1016/j.virusres.2021.198528.

12. Senpuku, K., Kataoka-Nakamura, C., Kunishima, Y., Hirai, T., and Yoshioka, Y. (2024). An inactivated whole-virion vaccine for Enterovirus D68 adjuvanted with CpG ODN or AddaVax elicits potent protective immunity in mice. Vaccine *42*, 2463–2474. https://doi.org/10.1016/j.vaccine.2024.03.016.

13. Senpuku, K., Kunishima, Y., Taniguchi, K., Ito, T., Hirai, T., Nakamura, T., Kataoka-Nakamura, C., and Yoshioka, Y. (2025). Development and characterization of MRC-5 and Vero cell-adapted enterovirus D68 strains for vaccine production. Vaccine *60*, 127314. https://doi.org/10.1016/j.vaccine.2025.127314.

14. Smee, D.F., Evans, W.J., Nicolaou, K.C., Tarbet, E.B., and Day, C.W. (2016). Susceptibilities of enterovirus D68, enterovirus 71, and rhinovirus 87 strains to various antiviral compounds. Antivir. Res. *131*, 61–65. https://doi.org/10.1016/j.antiviral.2016.04.003.

15. Gupta, R., Arora, K., Roy, S.S., Joseph, A., Rastogi, R., Arora, N.M., and Kundu, P.K. (2023). Platforms, advances, and technical challenges in virus-like particles-based vaccines. Front. Immunol. *14*, 1123805. https://doi.org/10.3389/fimmu.2023.1123805.

16. Jartti, M., Flodström-Tullberg, M., and Hankaniemi, M.M. (2024). Enteroviruses: epidemic potential, challenges and opportunities with vaccines. J. Biomed. Sci. *31*, 73. https://doi.org/10.1186/s12929-024-01058-x.

17. Ren, M., Abdullah, S.W., Pei, C., Guo, H., and Sun, S. (2024). Use of virus-like particles and nanoparticle-based vaccines for combating picornavirus infections. Vet. Res. *55*, 128. https://doi.org/10.1186/s13567-024-01383-x.

18. Bachmann, M.F., van Damme, P., Lienert, F., and Schwarz, T.F. (2025). Virus-like particles: a versatile and effective vaccine platform. Expert Rev. Vaccines *24*, 444–456. https://doi.org/10.1080/14760584.2025.2508517.

19. Dai, W., Zhang, C., Zhang, X., Xiong, P., Liu, Q., Gong, S., Geng, L., Zhou, D., and Huang, Z. (2018). A virus-like particle vaccine confers protection against enterovirus D68 lethal challenge in mice. Vaccine *36*, 653–659. https://doi.org/10.1016/j.vaccine.2017.12.057.

20. Zhang, C., Zhang, X., Zhang, W., Dai, W., Xie, J., Ye, L., Wang, H., Chen, H., Liu, Q., Gong, S., et al. (2018). Enterovirus D68 virus-like particles expressed in Pichia pastoris potently induce neutralizing antibody responses and confer protection against lethal viral infection in mice. Emerg. Microb. Infect. *7*, 1–22. https://doi.org/10.1038/s41426-017-0005-x.

21. Krug, P.W., Wang, L., Shi, W., Kong, W.P., Moss, D.L., Yang, E.S., Fisher, B.E., Morabito, K.M., Mascola, J.R., Kanekiyo, M., et al. (2023). EV-D68 virus-like particle vaccines elicit cross-clade neutralizing antibodies that inhibit infection and block dissemination. Sci. Adv. *9*, eadg6076. https://doi.org/10.1126/sciadv.adg6076.

22. Moss, D.L., Paine, A.C., Krug, P.W., Kanekiyo, M., and Ruckwardt, T.J. (2024). Enterovirus virus-like-particle and inactivated poliovirus vaccines do not elicit substantive cross-reactive antibody responses. PLoS Pathog. *20*, e1012159. https://doi.org/10.1371/journal.ppat.1012159.

23. Cheng, J., Krug, P.W., Lei, H., Moss, D.L., Lang, Z.C., Morton, A.J., Shen, C.H., Pletnev, S., Huang, R.K., Pierson, T.C., et al. (2025). Structural insights from vaccine candidates for EV-D68. Commun. Biol. *8*, 860. https://doi.org/10.1038/s42003-025-08253-y.

24. Rial, D.V., and Ceccarelli, E.A. (2002). Removal of DnaK contamination during fusion protein purifications. Protein Expr. Purif. *25*, 503–507. https://doi.org/10.1016/s1046-5928(02)00024-4.

25. Morales, E.S., Parcerisa, I.L., and Ceccarelli, E.A. (2019). A novel method for removing contaminant Hsp70 molecular chaperones from recombinant proteins. Protein Sci. *28*, 800–807. https://doi.org/10.1002/pro.3574.

26. Adeyemi, O.O., Nicol, C., Stonehouse, N.J., and Rowlands, D.J. (2017). Increasing Type 1 Poliovirus Capsid Stability by Thermal Selection. J. Virol. *91*, e0158616. https://doi.org/10.1128/JVI.01586-16.

27. Lin, Y.L., Hu, Y.C., Liang, C.C., Lin, S.Y., Liang, Y.C., Yuan, H.P., and Chiang, B.L. (2014). Enterovirus-71 virus-like particles induce the activation and maturation of human monocyte-derived dendritic cells through TLR4 signaling. PLoS One *9*, e111496. https://doi.org/10.1371/journal.pone.0111496.

28. Hoshino, K., Takeuchi, O., Kawai, T., Sanjo, H., Ogawa, T., Takeda, Y., Takeda, K., and Akira, S. (1999). Cutting edge: Toll-like receptor 4 (TLR4)-deficient mice are

hyporesponsive to lipopolysaccharide: evidence for TLR4 as the Lps gene product. J. Immunol. *162*, 3749–3752.

29. Dai, W., Li, X., Liu, Z., and Zhang, C. (2023). Identification of four neutralizing antigenic sites on the enterovirus D68 capsid. J. Virol. *97*, e0160023. https://doi.org/10.1128/jvi.01600-23.
30. Liu, Y., Sheng, J., Fokine, A., Meng, G., Shin, W.H., Long, F., Kuhn, R.J., Kihara, D., and Rossmann, M.G. (2015). Structure and inhibition of EV-D68, a virus that causes respiratory illness in children. Science *347*, 71–74. https://doi.org/10.1126/science.1261962.
31. Liu, Y., Sheng, J., van Vliet, A.L.W., Buda, G., van Kuppeveld, F.J.M., and Rossmann, M.G. (2018). Molecular basis for the acid-initiated uncoating of human enterovirus D68. Proc. Natl. Acad. Sci. USA *115*, E12209–E12217. https://doi.org/10.1073/pnas.1803347115.
32. Zheng, Q., Zhu, R., Xu, L., He, M., Yan, X., Liu, D., Yin, Z., Wu, Y., Li, Y., Yang, L., et al. (2018). Atomic structures of enterovirus D68 in complex with two monoclonal antibodies define distinct mechanisms of viral neutralization. Nat. Microbiol. *4*, 124–133. https://doi.org/10.1038/s41564-018-0275-7.
33. Hipp, M.S., Kasturi, P., and Hartl, F.U. (2019). The proteostasis network and its decline in ageing. Nat. Rev. Mol. Cell Biol. *20*, 421–435. https://doi.org/10.1038/s41580-019-0101-y.
34. Geeraedts, F., Goutagny, N., Hornung, V., Severa, M., de Haan, A., Pool, J., Wilschut, J., Fitzgerald, K.A., and Huckriede, A. (2008). Superior immunogenicity of inactivated whole virus H5N1 influenza vaccine is primarily controlled by Toll-like receptor signalling. PLoS Pathog. *4*, e1000138. https://doi.org/10.1371/journal.ppat.1000138.
35. Koyama, S., Aoshi, T., Tanimoto, T., Kumagai, Y., Kobiyama, K., Tougan, T., Sakurai, K., Coban, C., Horii, T., Akira, S., and Ishii, K.J. (2010). Plasmacytoid dendritic cells delineate immunogenicity of influenza vaccine subtypes. Sci. Transl. Med. *2*, 25ra24. https://doi.org/10.1126/scitranslmed.3000759.
36. Ponterio, E., Mariotti, S., Tabolacci, C., Ruggeri, F.M., and Nisini, R. (2019). Virus like particles of GII.4 norovirus bind Toll Like Receptors 2 and 5. Immunol. Lett. *215*, 40–44. https://doi.org/10.1016/j.imlet.2019.05.016.
37. Liu, Y., Sheng, J., Baggen, J., Meng, G., Xiao, C., Thibaut, H.J., van Kuppeveld, F.J.M., and Rossmann, M.G. (2015). Sialic acid-dependent cell entry of human enterovirus D68. Nat. Commun. *6*, 8865. https://doi.org/10.1038/ncomms9865.
38. Pereirinha da Silva, A.K., van Trijp, J.P., Montenarie, A., Fok, J.A., Sooksawasdi Na Ayudhya, S., Pieters, R.J., Boons, G.J., van Riel, D., de Vries, R.P., and Bauer, L. (2025). Sialic Acid-Containing Glycolipids Extend the Receptor Repertoire of Enterovirus-D68. ACS Infect. Dis. *11*, 2090–2103. https://doi.org/10.1021/acsinfecdis.5c00063.
39. Liu, X., Li, H., Li, Z., Gao, D., Zhou, J., Ni, F., Yu, Q., Huang, Y., Tang, Y., Xue, L., et al. (2025). MFSD6 is an entry receptor for respiratory enterovirus D68. Cell Host Microbe *33*, 267–278.e4. https://doi.org/10.1016/j.chom.2024.12.015.
40. Varanese, L., Xu, L., Peters, C.E., Pintilie, G., Roberts, D.S., Raj, S., Liu, M., Ooi, Y.S., Diep, J., Qiao, W., et al. (2025). MFSD6 is an entry receptor for enterovirus D68. Nature *641*, 1268–1275. https://doi.org/10.1038/s41586-025-08908-0.
41. Jayawardena, N., McCarthy, C., Wang, I., Waqqar, S., Burga, L.N., Strauss, M., and Bostina, M. (2022). Characterisation of a Seneca Valley virus thermostable mutant. Virology *575*, 74–82. https://doi.org/10.1016/j.virol.2022.08.015.
42. Sherry, L., Bahar, M.W., Porta, C., Fox, H., Grehan, K., Nasta, V., Duyvesteyn, H.M.E., De Colibus, L., Marsian, J., Murdoch, I., et al. (2025). Recombinant expression systems for production of stabilised virus-like particles as next-generation polio vaccines. Nat. Commun. *16*, 831. https://doi.org/10.1038/s41467-025-56118-z.
43. Kyo, F., Futani, H., Matsui, K., Terada, M., Adachi, K., Nagata, K., Sano, H., Tateishi, H., Tsutsui, H., and Nakanishi, K. (2005). Endogenous interleukin-6, but not tumor necrosis factor alpha, contributes to the development of toll-like receptor 4/myeloid differentiation factor 88-mediated acute arthritis in mice. Arthritis Rheum. *52*, 2530–2540. https://doi.org/10.1002/art.21213.
44. Sun, S., Gao, F., Hu, Y., Bian, L., Wu, X., Su, Y., Du, R., Fu, Y., Zhu, F., Mao, Q., and Liang, Z. (2018). A cross-sectional seroepidemiology study of EV-D68 in China. Emerg. Microb. Infect. *7*, 1–7. https://doi.org/10.1038/s41426-018-0103-4.
45. Mastronarde, D.N. (2005). Automated electron microscope tomography using robust prediction of specimen movements. J. Struct. Biol. *152*, 36–51. https://doi.org/10.1016/j.jsb.2005.07.007.
46. Punjani, A., Rubinstein, J.L., Fleet, D.J., and Brubaker, M.A. (2017). cryoSPARC: algorithms for rapid unsupervised cryo-EM structure determination. Nat. Methods *14*, 290–296. https://doi.org/10.1038/nmeth.4169.
47. Croll, T.I. (2018). ISOLDE: a physically realistic environment for model building into low-resolution electron-density maps. Acta Crystallogr. D Struct. Biol. *74*, 519–530. https://doi.org/10.1107/S2059798318002425.
48. Meng, E.C., Goddard, T.D., Pettersen, E.F., Couch, G.S., Pearson, Z.J., Morris, J.H., and Ferrin, T.E. (2023). UCSF ChimeraX: Tools for structure building and analysis. Protein Sci. *32*, e4792. https://doi.org/10.1002/pro.4792.

OMTN, Volume *37*

Supplemental information

Comparative immunogenic and structural analysis of virus-like particle and inactivated whole-virion vaccines against enterovirus D68

Kota Senpuku, Yuta Kunishima, Mika Hirose, Tatsuya Karaki, Kotaro Taniguchi, Chikako Kataoka-Nakamura, Toshiro Hirai, Koubun Yasuda, Etsushi Kuroda, Takayuki Kato, Taiki Ito, and Yasuo Yoshioka

A

	Database	Accession	Score	Mass	Num. of matches	Num. of significant matches	Num. of sequences	Num. of significant sequences	emPAI	Description
**1**	SwissProt	HS90B_HUMAN	4632	83554	201	201	45	45	12.84	Heat shock protein HSP 90-beta
**2**	SwissProt	HS90A_HUMAN	4315	85006	196	196	43	43	9.02	Heat shock protein HSP 90-alpha
**3**	SwissProt	HS71A_HUMAN	1461	70294	70	70	19	19	2.48	Heat shock 70 kDa protein 1A
**4**	SwissProt	EF2_HUMAN	848	96246	55	55	22	22	1.77	Elongation factor 2
**5**	SwissProt	IMB1_HUMAN	785	98420	39	39	16	16	1.28	Importin subunit beta-1
**6**	SwissProt	K2C1_HUMAN	623	66170	32	32	16	16	1.92	Keratin, type II cytoskeletal 1
**7**	SwissProt	UBA1_HUMAN	590	118858	31	31	14	14	0.62	Ubiquitin-like modifier-activating enzyme 1
**8**	SwissProt	TNPO1_HUMAN	536	103771	29	29	14	14	0.63	Transportin-1
**9**	SwissProt	MCM5_HUMAN	492	83031	31	31	18	18	1.26	DNA replication licensing factor MCM5
**10**	SwissProt	MCM2_HUMAN	481	102516	32	32	17	17	0.93	DNA replication licensing factor MCM2

B

	Database	Accession	Score	Mass	Num. of matches	Num. of significant matches	Num. of sequences	Num. of significant sequences	emPAI	Description
**1**	SwissProt	HS71A_HUMAN	7451	70294	350	350	38	38	25.78	Heat shock 70 kDa protein 1A
**2**	SwissProt	HSP7C_HUMAN	2064	71082	106	106	23	23	6.1	Heat shock cognate 71 kDa protein
**3**	SwissProt	HSP72_HUMAN	1160	70263	53	53	13	13	1.27	Heat shock-related 70 kDa protein 2
**4**	SwissProt	XRCC6_HUMAN	788	70084	40	40	16	16	1.28	X-ray repair cross-complementing protein 6
**5**	SwissProt	PPCE_HUMAN	727	81560	43	43	21	21	2.08	Prolyl endopeptidase
**6**	SwissProt	HS90B_HUMAN	699	83554	39	39	19	19	1.45	Heat shock protein HSP 90-beta
**7**	SwissProt	K2C1_HUMAN	664	66170	34	34	15	15	1.65	Keratin, type II cytoskeletal 1
**8**	SwissProt	SYRC_HUMAN	659	76129	41	41	22	22	2.19	Arginine--tRNA ligase, cytoplasmic
**9**	SwissProt	AMPB_HUMAN	588	73234	32	32	17	17	1.2	Aminopeptidase B
**10**	SwissProt	SNX2_HUMAN	581	58549	36	36	15	15	2.38	Sorting nexin-2

Figure S1. Identification of proteins via LC-MS/MS analysis. LC-MS/MS analysis was performed on the protein bands marked in red in Figure 1C, approximately corresponding to (A) 90 kDa and (B) 70 kDa. The table lists the top 10 candidate proteins identified. emPAI: Exponentially modified protein abundance Index.

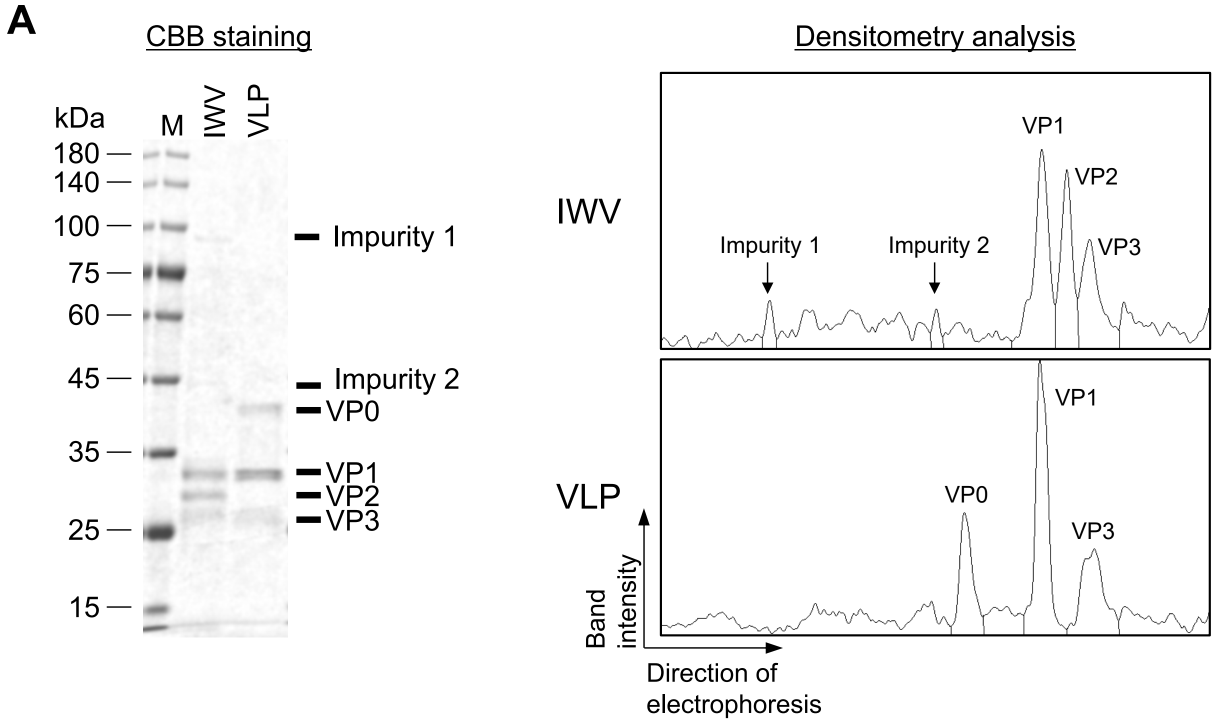

B

IWV		VLP	
Band	Intensity	Band	Intensity
VP1	5759	VP1	6524
VP2	4022	VP0	3104
VP3	3920	VP3	3756
Impurity 1	711		
Impurity 2	552		
Total	14964	Total	13384
VP1-3 total	13701	V0-3 total	13384
Purity	91.5%	Purity	100%

Figure S2. Purity of IWV and VLP used for the vaccination. (A) Purified IWV and VLP (1 μg/lane) were subjected to SDS-PAGE followed by Coomassie Brilliant Blue (CBB) staining. The picture of the gel after CBB staining is shown in the left panel. The detected bands are labeled accordingly. M indicates molecular size marker. The results of the quantitative densitometric analysis for each lane are shown in the right panel. (B) The table showing the result of the band quantification based on the result of the densitometry analysis (right panel of A). Data are representative of at least two independent preparations.

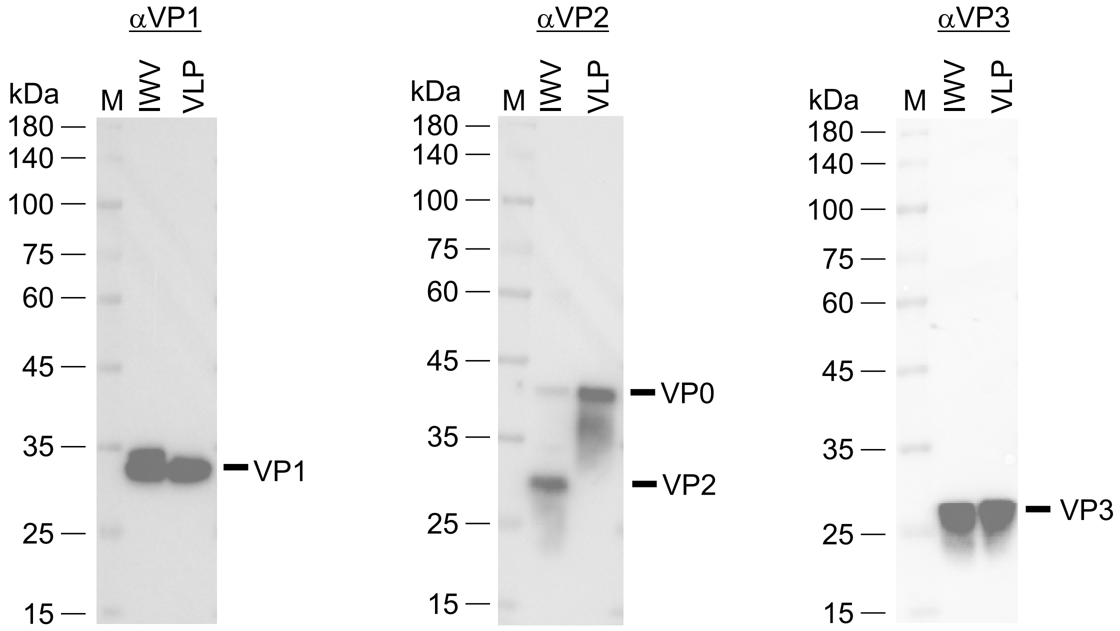

Figure S3. Western blot analysis of purified IWV and VLP. Purified IWV and VLP samples (50 ng/lane) were subjected to western blotting and probed with antibodies specific to VP1, VP2, and VP3 to confirm the presence of capsid proteins. M indicates molecular size marker.

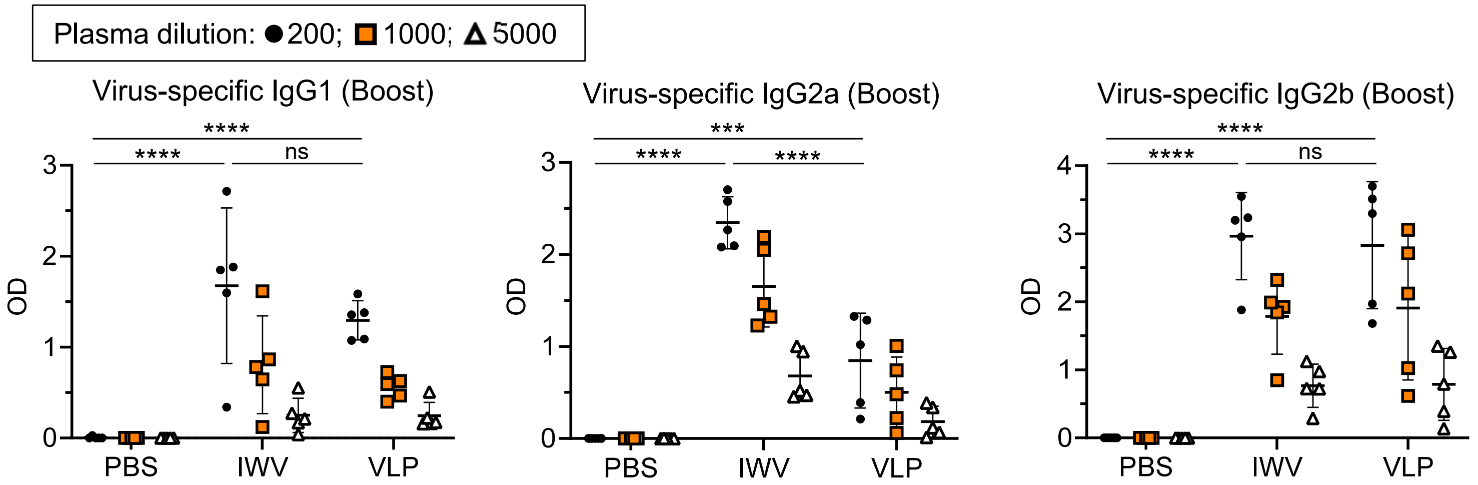

Figure S4. IgG subclass profiles of EV-D68-specific antibodies induced by IWV and VLP. Post-boost plasma levels of virus-specific IgG1, IgG2a, and IgG2b were measured. Data represent the mean ± SD (n = 5 per group). Statistical analyses were performed using 200-fold-diluted plasma samples. "ns" indicates no significant difference. ***$P < 0.001$; ****$P < 0.0001$ as determined by Tukey's test.

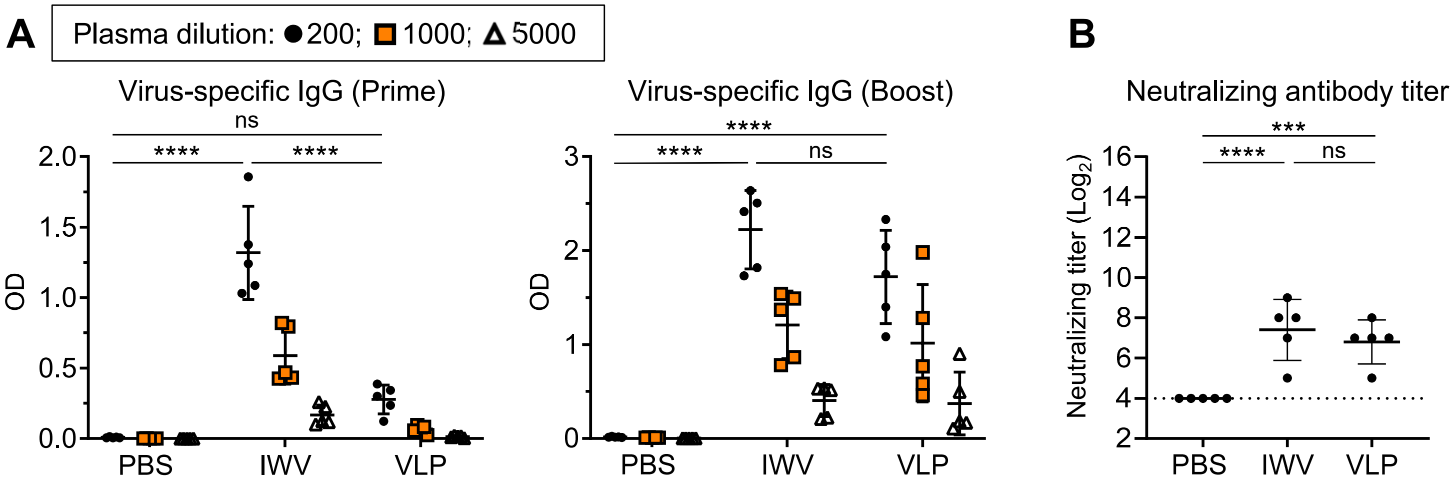

Figure S5. Immunogenicity of IWV and VLP vaccines following intramuscular immunization. Female BALB/c mice were immunized intramuscularly with 0.1 µg of either IWV or VLP on days 0 and 21. (A) Plasma IgG levels specific to IWV following prime and boost immunization. (B) Neutralizing antibody titers against the MO strain measured after boost immunization. (A, B) Each group consisted of n = 5 mice. Data are presented as mean ± SD. (B) Dotted lines represent the limit of detection. (A) Statistical analyses were performed using plasma diluted 1:200. (A, B) "ns" indicates not significant. ***$P < 0.001$; ****$P < 0.0001$, as determined by Tukey's test.

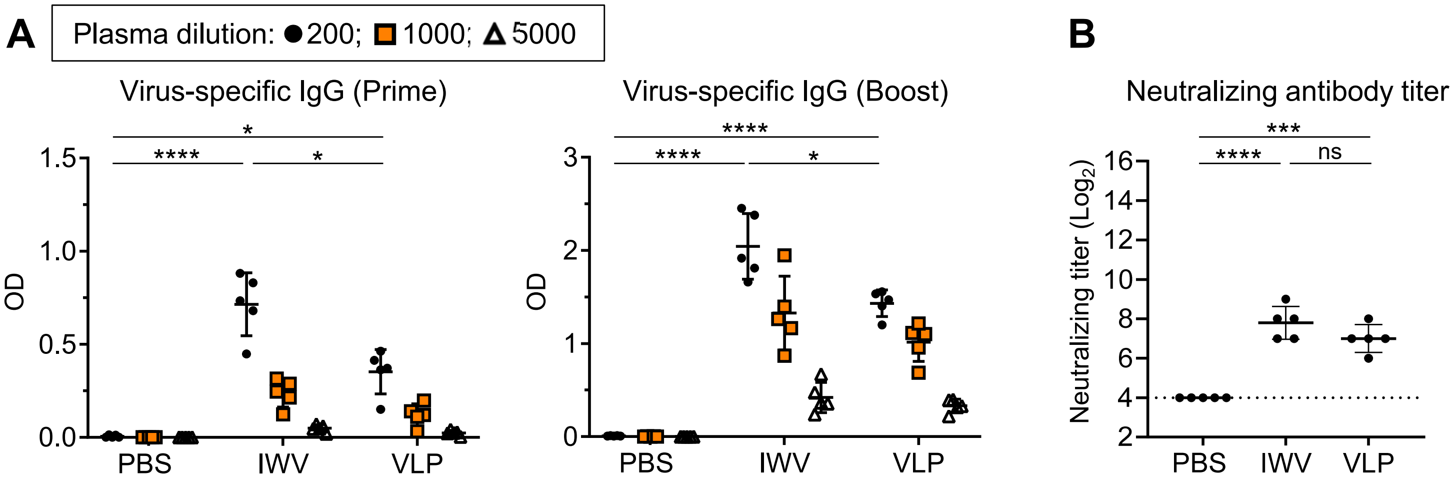

Figure S6. Immunogenicity of IWV and VLP vaccines in male BALB/c mice. Male BALB/c mice were immunized subcutaneously with 0.1 μg of either IWV or VLP on days 0 and 21. (A) Plasma IgG levels specific to IWV following prime and boost immunization. (B) Neutralizing antibody titers against the MO strain measured after boost immunization. (A, B) Each group consisted of n = 5 mice. Data are presented as mean ± SD. (B) Dotted lines represent the limit of detection. (A) Statistical analyses were performed using plasma diluted 1:200. (A, B) "ns" indicates not significant. **P* < 0.05; ****P* < 0.001; *****P* < 0.0001, as determined by Tukey's test.

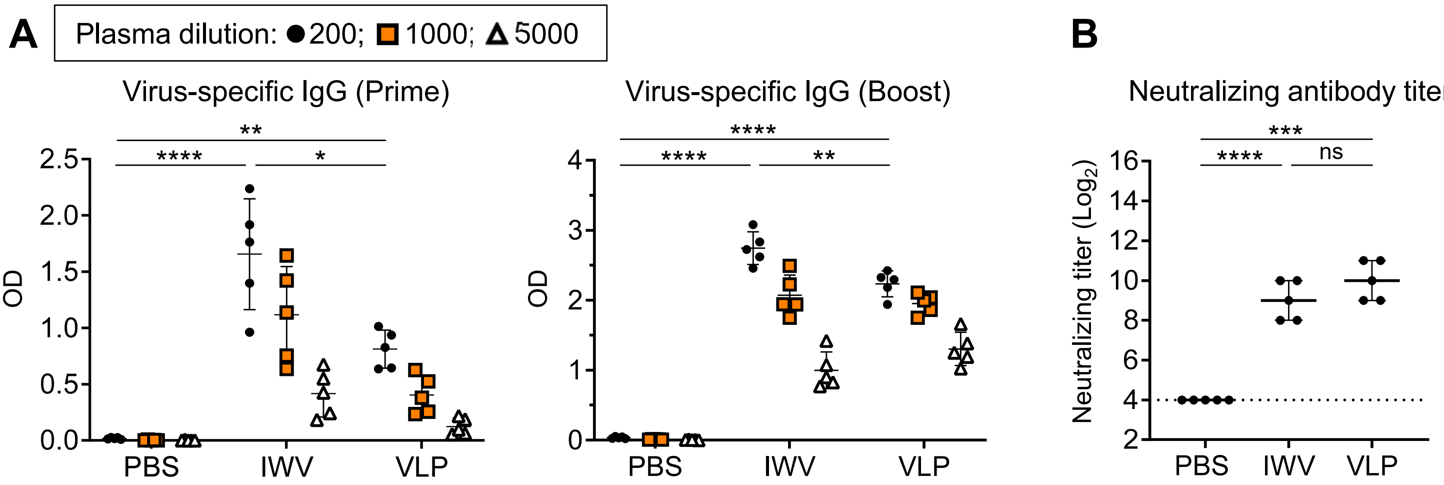

Figure S7. Immunogenicity of IWV and VLP vaccines in female CB6F1 mice. Female CB6F1 mice were immunized subcutaneously with 0.1 μg of either IWV or VLP on days 0 and 21. (A) Plasma IgG levels specific to IWV following prime and boost immunization. (B) Neutralizing antibody titers against the MO strain measured after boost immunization. (A, B) Each group consisted of n = 5 mice. Data are presented as mean ± SD. (B) Dotted lines represent the limit of detection. (A) Statistical analyses were performed using plasma diluted 1:200. (A, B) "ns" indicates not significant. **P* < 0.05; ***P* < 0.01; ****P* < 0.001; *****P* < 0.0001, as determined by Tukey's test.

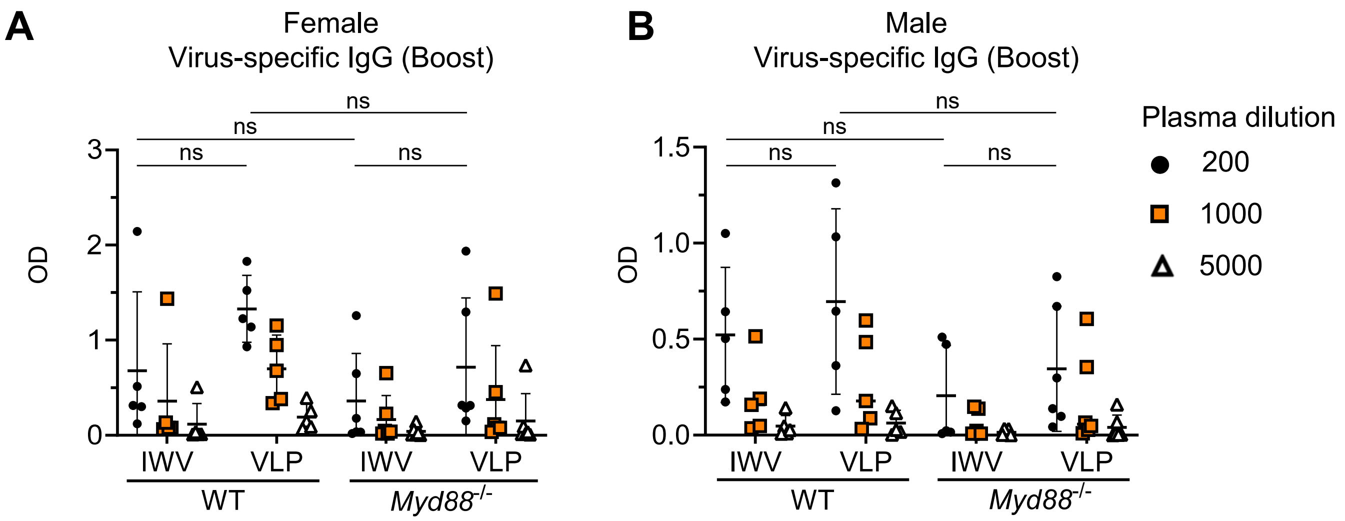

Figure S8. MyD88-independent immune responses induced by IWV and VLP vaccines. (A) Female and (B) male wild-type (WT) and MyD88-deficient (*Myd88*$^{-/-}$) mice were immunized subcutaneously with 0.1 μg of IWV or VLP on days 0 and 21. Virus-specific IgG levels in plasma from (A) female and (B) male mice following boost immunization were shown. (A, B) Each group consisted of n = 5 or 6 mice. Data are presented as mean ± SD. Statistical analyses were performed using plasma diluted 1:200. "ns" indicates not significant, as determined by Tukey's test.

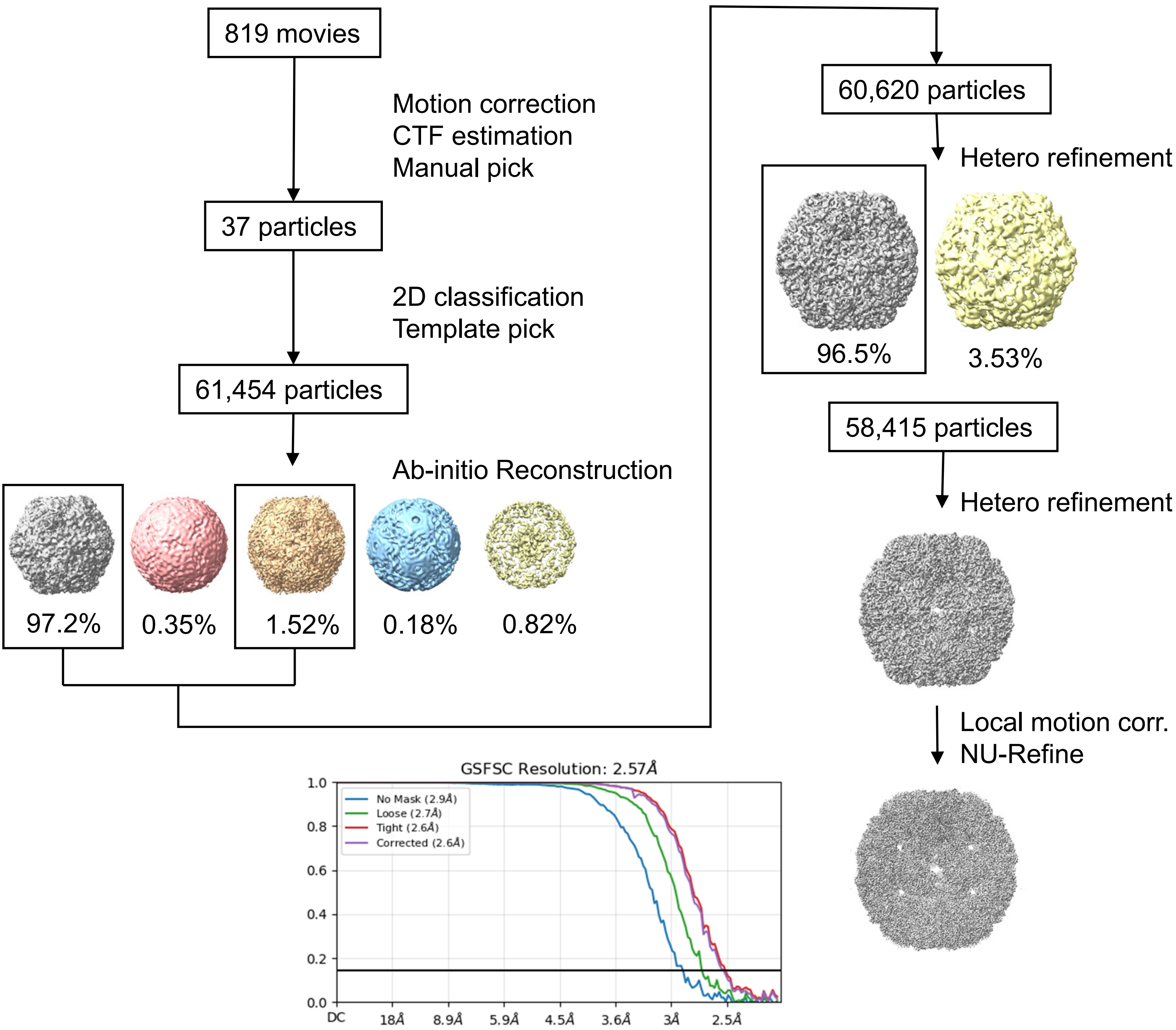

Figure S9. Workflow of cryo-EM image processing. Overall workflow of image processing using cryoSPARC version 4.6.0 software. Gold-standard Fourier shell correlation curves of the reconstructed cryo-EM maps are shown in the bottom.

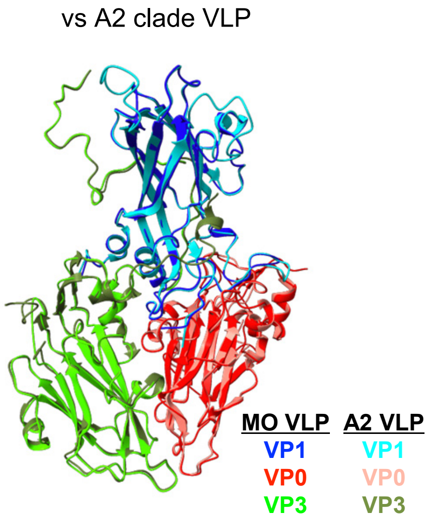

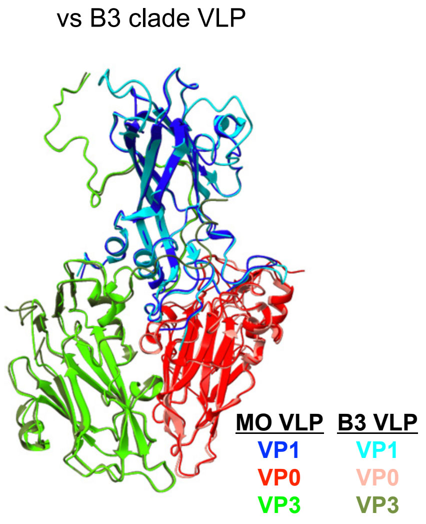

vs A2 clade VLP	Pruned atom pairs	RMSD (Å)
VP1	190	0.459
VP0	186	0.477
VP3	218	0.455
vs B3 clade VLP	Pruned atom pairs	RMSD (Å)
VP1	189	0.438
VP0	187	0.608
VP3	218	0.491

Figure S10. Structural Comparison of VLPs from multiple strains. A structural comparison was performed between the icosahedral asymmetric unit of the VLP from the MO strain (PDB: 9W4I) and those from the A2 subclade (left; PDB: 9C4A) and the B3 subclade (middle; PDB: 9C3J). VP1, VP0, and VP3 subunits of each VLP were superimposed onto the corresponding subunits of the mature virion and the empty particle. Representative structures are shown based on superposition using VP1 as the reference. All structures are displayed as cartoon models. The table in the right panel summarizes the number of pruned atom pairs and root mean square deviation (RMSD) values for each superposition.

A

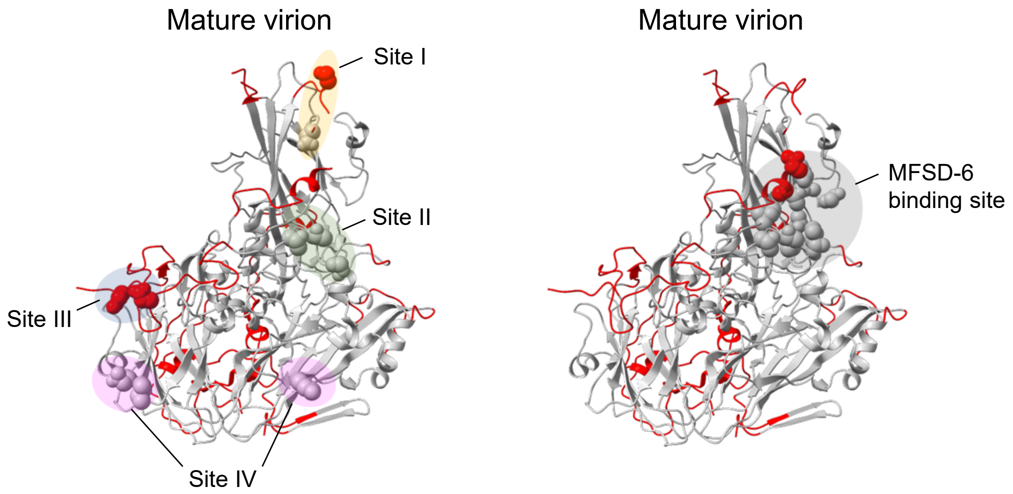

B

VP4 (VP0 in VLP)

1 10 20 30 40 50 60
Mature virion: MGAQVTRQQTGTHENANIATNGSHITYNQINFYKDSYAASASKQDFSQDPSKFTEPVVEGLKAGAPVLK
VLP: MGAQVTRQQTGTHENANIATNGSHITYNQINFYKDSYAASASKQDFSQDPSKFTEPVVEGLKAGAPVLK

VP2 (VP0 in VLP)

1 10 20 30 40 50 60 70
Mature virion: SPSAEACGYSDRVLQLKLGNSAIVTQEAANYCCAYGEWPNYLPDHEAVAIDKPTQPETATDRFYTLKSVK
VLP: SPSAEACGYSDRVLQLKLGNSAIVTQEAANYCCAYGEWPNYLPDHEAVAIDKPTQPETATDRFYTLKSVK

71 80 90 100 110 120 130 140
Mature virion: WETGSTGWWWKLPDALNNIGMFGQNVQHHYLYRSGFLIHVQCNATKFHQGALLVVAIPEHQRGAHNTNTS
VLP: WETGSTGWWWKLPDALNNIGMFGQNVQHHYLYRSGFLIHVQCNATKFHQGALLVVAIPEHQRGAHNTNTS

141 150 160 170 180 190 200 210
Mature virion: PGFDDIMKGEEGGTFNHPYVLDDGTSLACATIFPHQWINLRTNNSATIVLPWMNAAPMDFPLRHNQWTLA
VLP: PGFDDIMKGEEGGTFNHPYVLDDGTSLACATIFPHQWINLRTNNSATIVLPWMNAAPMDFPLRHNQWTLA

211 220 230 240
Mature virion: IIPVVPLGTRTTSSMVPITVSIAPMCCEFNGLRHAITQ
VLP: IIPVVPLGTRTTSSMVPITVSIAPMCCEFNGLRHAITQ

VP3

1 10 20 30 40 50 60 70
Mature virion: GVPTYLLPGSGQFLTTDDHSSAPALPCFNPTPEMHIPGQVRNMLEVVQVESMMEINNTESAVGMERLKVD
VLP: GVPTYLLPGSGQFLTTDDHSSAPALPCFNPTPEMHIPGQVRNMLEVVQVESMMEINNTESAVGMERLKVD

71 80 90 100 110 120 130 140
Mature virion: ISALTDVDQLLFNIPLDIQLDGPLRNTLVGNISRYYTHWSGSLEMTFMFCGSFMAAGKLILCYTPPGGSC
VLP: ISALTDVDQLLFNIPLDIQLDGPLRNTLVGNISRYYTHWSGSLEMTFMFCGSFMAAGKLILCYTPPGGSC

141 150 160 170 180 190 200 210
Mature virion: PTTRETAMLGTHIVWDFGLQSSVTLIIPWISGSHYRMFNNDAKSTNANVGYVTCFMQTNLIVPSESSDTC
VLP: PTTRETAMLGTHIVWDFGLQSSVTLIIPWISGSHYRMFNNDAKSTNANVGYVTCFMQTNLIVPSESSDTC

211 220 230 240
Mature virion: SLIGFIAAKDDFSLRLMRDSPDIGQLDHLHAAEAAYQ
VLP: SLIGFIAAKDDFSLRLMRDSPDIGQLDHLHAAEAAYQ

VP1

1 10 20 30 40 50 60 70
Mature virion: IESIIKTATDTVKSEINAELGVVPSLNAVETGATSNTEPEEAIQTRTVINQHGVSETLVENFLSRAALVS
VLP: IESIIKTATDTVKSEINAELGVVPSLNAVETGATSNTEPEEAIQTRTVINQHGVSETLVENFLSRAALVS

71 80 90 100 110 120 130 140
Mature virion: KRSFEYKDHTSSTARADKNFFKWTINTRSFVQLRRKLELFTYLRFDAEITILTTVAVNGSGNNTYVGLPD
VLP: KRSFEYKDHTSSTARADKNFFKWTINTRSFVQLRRKLELFTYLRFDAEITILTTVAVNGSGNNTYVGLPD

141 150 160 170 180 190 200 210
Mature virion: LTLQAMFVPTGALTPEKQDSFHWQSGSNASVFFKISDPPARITIPFMCINSAYSVFYDGFAGFEKNGLYG
VLP: LTLQAMFVPTGALTPEKQDSFHWQSGSNASVFFKISDPPARITIPFMCINSAYSVFYDGFAGFEKNGLYG

211 220 230 240 250 260 270 280
Mature virion: INPADTIGNLCVRIVNEHQPVGFTVTVRVYMKPKHIKAWAPRPPRTLPYMSIANANYKGKERAPNALSAI
VLP: INPADTIGNLCVRIVNEHQPVGFTVTVRVYMKPKHIKAWAPRPPRTLPYMSIANANYKGKERAPNALSAI

281 290
IGNRDSVKTMPHNIVNT
IGNRDSVKTMPHNIVNT

Figure S11. Comparison of structurally resolved regions between the mature virion and VLP. (A) Regions that are structurally unresolved in the VLP are highlighted in red on the mature virion structure, displayed as a cartoon model. Antigenic sites are represented as spheres in the left panel and are color-coded according to Figure 4A. MFSD-6 interaction sites are shown as spheres in the right panel. (B) Amino acid sequences of the structural protein subunits (VP4, VP2, VP3, and VP1) were aligned. Structurally resolved regions are shown in black, whereas unresolved (disordered or flexible) regions are depicted in gray. Structural information was obtained from cryo-EM models with the following PDB IDs: 6CRU (mature virion) and 9W4I (VLP).

Table S1. Cryo-EM data collection, refinement, and validation statistics.

	VLP MO strain (EMDB-65634) (PDB 9W4I)
Data collection and processing	
Magnification (Nominal)	81 000
Voltage (kV)	300
Electron exposure (e–/Å^2)	50
Defocus range (μm)	0.11 to 2.59
Pixel size (Å)	1.11
Symmetry imposed	I
Initial particle images (no.)	274 941
Final particle images (no.)	58 404
Map resolution (Å)	2.57
FSC threshold	0.143
Map resolution range (Å)	1.14-3.58
Refinement	
Initial model used	in silico model
Model composition	
Nonhydrogen atoms	4873
B factors (Å^2)	
Protein	84.62
R.m.s. deviations	
Bond lengths (Å)	0.012
Bond angles (°)	1.934
Validation	
MolProbity score	0.58
Clashscore	0.10
Poor rotamers (%)	0.93
Ramachandran plot	
Favored (%)	97.85
Allowed (%)	2.15
Disallowed (%)	0.00
